# Supplementary material for: Selenoprotein deficiency disorder predisposes to aortic aneurysm formation
Source: Nat Commun. 2023 Dec 2;14:7994. doi: 10.1038/s41467-023-43851-6 (PMC10693596; doi:10.1038/s41467-023-43851-6)

Supplementary Information

Selenoprotein deficiency disorder predisposes to aortic aneurysm formation

Supplementary Figure 1: *SECISBP2* genotype and biochemical phenotype in patient P1 and family members.

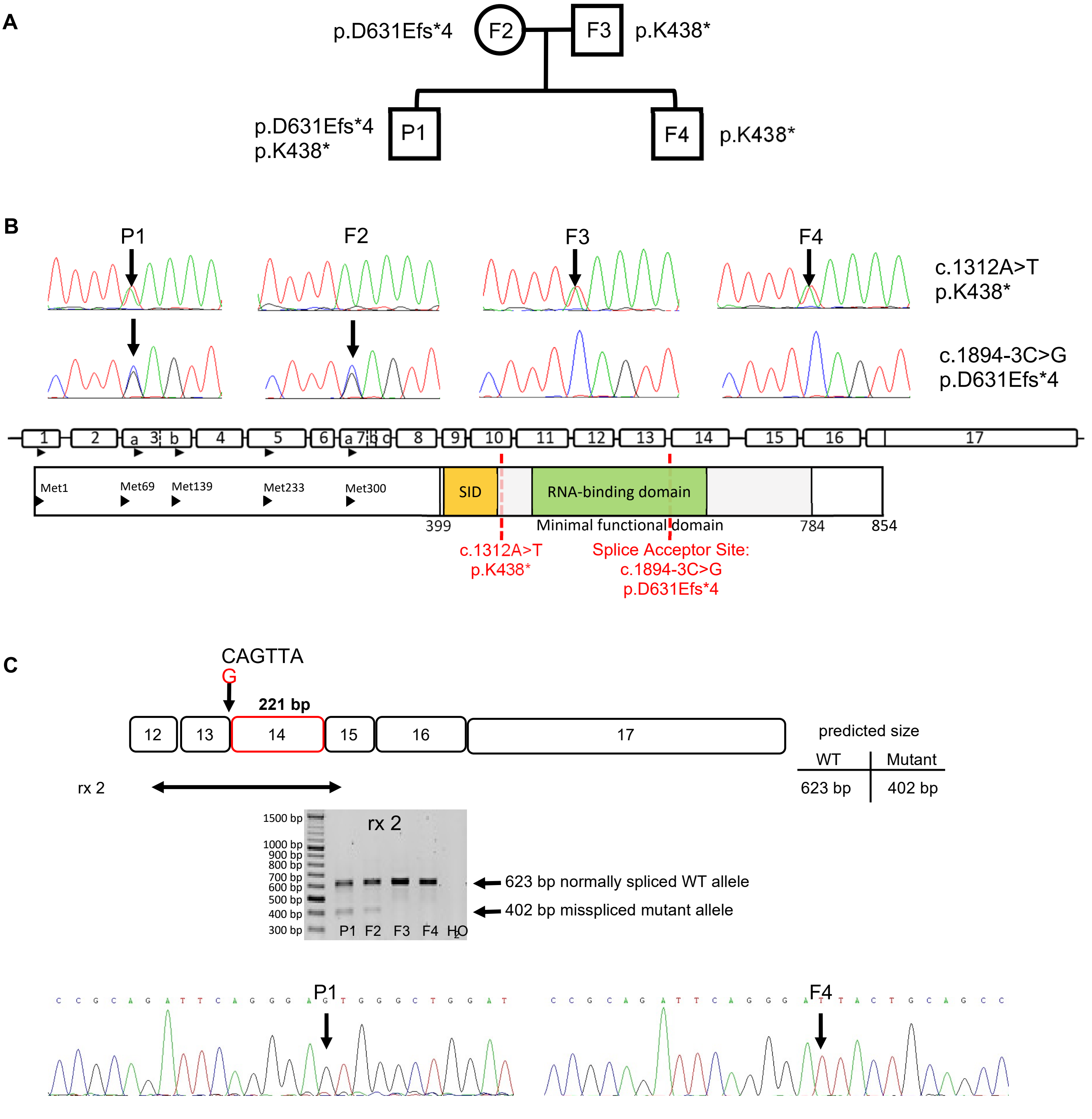

D

	P1	F2	F3	F4	Normal range
**TSH**	2,1	2,17	0,421	1,02	0,4-4,3 mU/L
**T4**	244	85	91	108	58-128 nmol/L
**FT4**	46	12,3	12,3	16,1	11-25 pmol/L
**T3**	1,22	1,82	2,38	2,33	1,4-2,5 nmol/L
**rT3**	1,1	0,27	0,44	0,4	0,22-0,59 nmol/L
**T3/rT3**	1,1	6,7	5,4	5,8	3-13
**SHBG**	78,6	56,8	57,4	36	10-70 mmol/L
**Se**	0.23	1.14	0.93	0.84	0,8-2,0 umol/L

A. Family pedigree of patient P1, squares and circles represent male and female family members (F2-4), respectively.
B. Electropherograms showing compound heterozygosity for two nucleotide substitutions: A to T in exon 10 and C to G in the intron between exon 13-14, indicated by arrows. Schematic representation of *SECISBP2* coding exons and its protein structure, with ATG codons (triangles) which could function as alternative translation initiation sites shown, with Met1 yielding full-length wild-type SECISBP2 protein. The minimal functional protein, located between amino acids 399 and 784, contains the Sec incorporation domain (SID) and RNA-binding domain. The location of mutations (c.1312 A>T, p.K438*; c.1894-3C>G, p.D631Efs*4) is superimposed.
C. Top. Genomic region of *SECISBP2*, showing position of the splice site mutation and affected exon 14 in red. **Middle**. Results of RT-PCR of aberrantly spliced *SECISBP2* mRNA transcripts, amplified from fibroblasts from Patient P1 and family members (F2, F3, F4), using a rx2 (forward in exon 12, reverse in exon 14) primer set, identifying a 623bp band corresponding to the correct splicing of the wild-type allele over exons 12-13-14-15 and a 402bp band resulting from mis-splicing of the mutated allele, lacking exon 14. Translation of the aberrantly spliced variant results in a frameshift and predicted premature stop at amino acid position 631. **Bottom**. Electropherograms showing incorrect spliced mRNA of patient P1 and correct sequence in brother F4.
D. Circulating thyroid hormones and biochemistry (TSH, thyroid stimulating hormone; T4, total thyroxine; FT4, free thyroxine; T3, total triiodothyronine; rT3, reverse triiodothyronine; SHBG, sex hormone binding globulin; Se, selenium) in patient P1 and family members (F2-4). High values are shaded red and low values blue.

Supplementary Figure 2: Selenoprotein deficiency in aortic vascular smooth muscle cells (VSMCs) from patients P1 and P2.

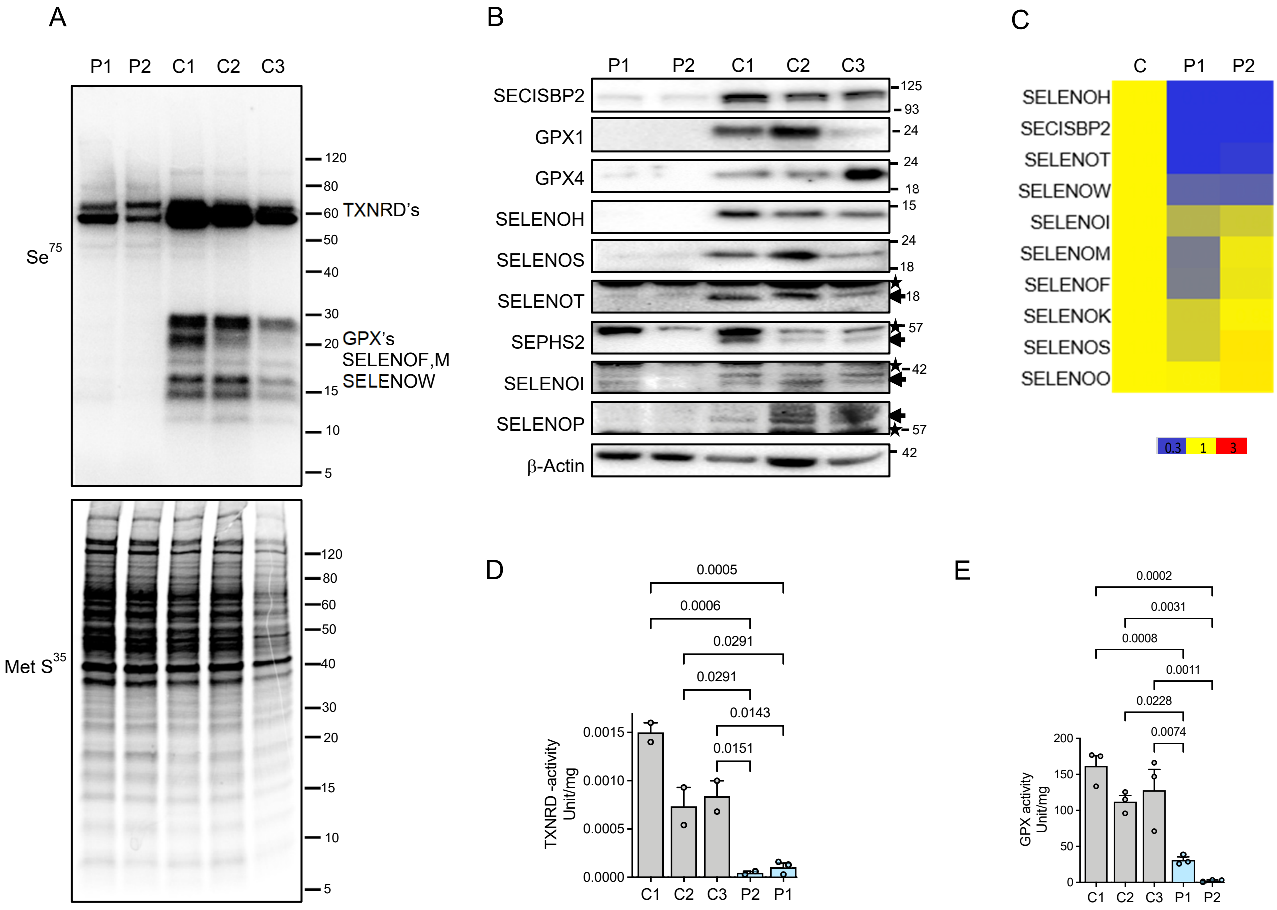

A. Selenoprotein biosynthesis in VSMCs from controls (C1, C2, C3) and patients P1 and P2, assessed using ^{75}Se labelling (upper panel). ^{35}S-Met labelling confirmed comparable protein loading (lower panel). Representative image of three independent experiments.

B. Western blotting showing reduced selenoprotein expression in primary VSMCs from Patients P1 and P2 compared to three controls (C1, C2, C3), with β-Actin as loading control. Arrows and stars denote specific and non-specific bands respectively. n=1 experiment.

C. Heat map of relative mRNA levels of several selenoproteins and *SECISBP2* in VSMCs from control subjects (C) and patient P1 and P2 measured by qPCR. Red indicates higher (max3x) and blue (0.3x) lower levels of gene expression relative to the control (uniformly designated yellow). P1 n=3; P2 n=2; C1 n=2; C2 n=2; C3 n=3 independent experiments.

D. Glutathione peroxidase activity in VSMCs from P1, P2 compared to healthy controls (C1, C2, C3). Three independent experiments.

E. Thioredoxin reductase activity in VSMCs from P1, P2 compared to healthy controls (C1, C2, C3).

*p<0.05 comparing patients with controls. Two independent experiments.

D-E. Statistics: ordinary one-way ANOVA with adjusted P values (Tukey's multiple comparison test), each bar represents the mean value, error bars represent SEM.

Source data are provided as a Source data file.

Supplementary Figure 3: Selenoprotein deficiency in patient P3.

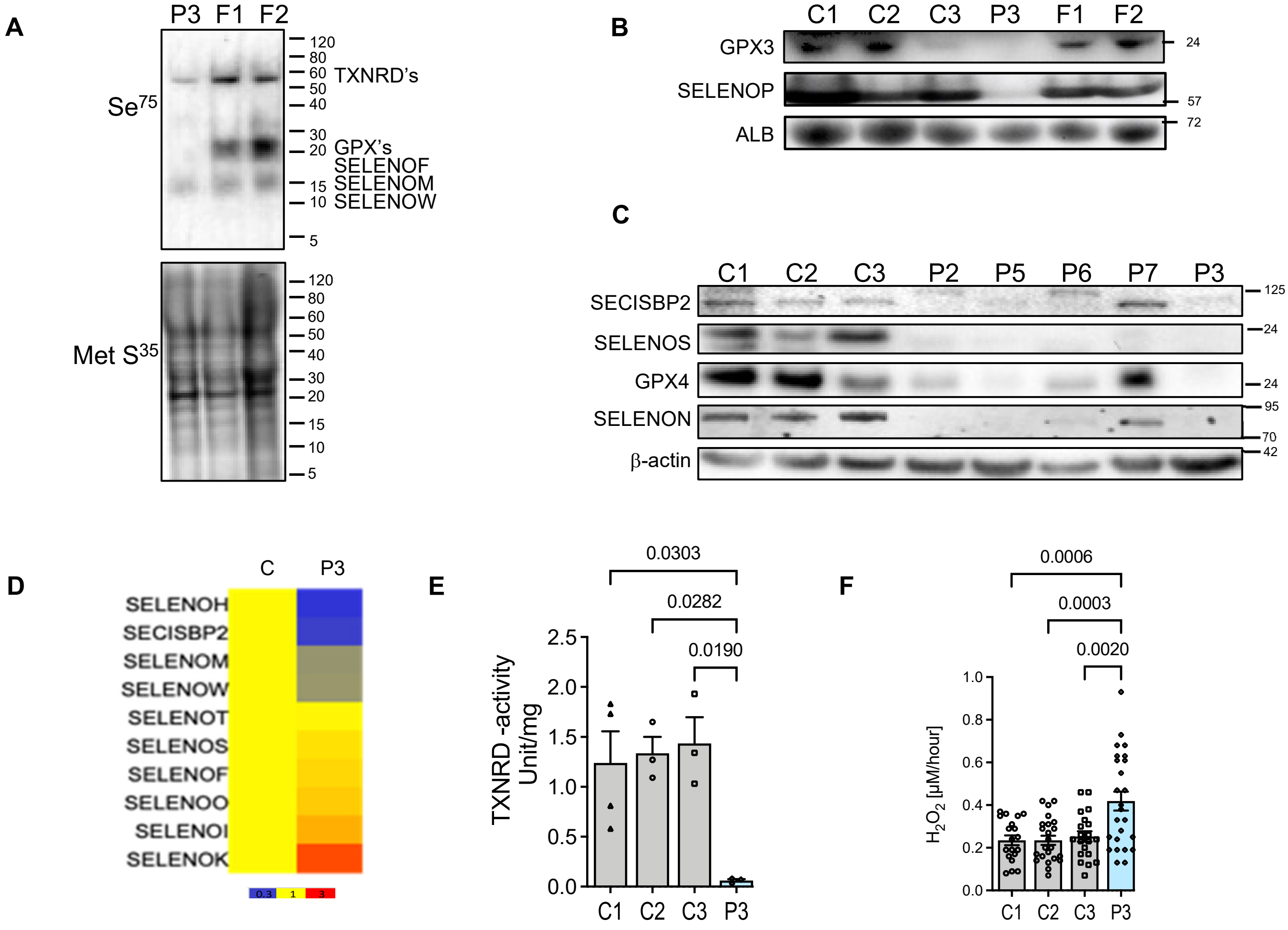

A. ^{75}Se labelling of peripheral blood mononuclear cells from patient P3 compared to family members (F1, F2) (upper panel). ^{35}S-Met labelling confirmed comparable protein loading (lower panel). n=1 experiment.

B. Western blot of serum selenoproteins (GPX3, SELENOP) from patient P3, family members (F1, F2) and three unrelated controls, with albumin as loading control. Representative image of two experiment.

C. Western blot of selenoproteins in dermal fibroblasts from control subjects (C1, C2, C3), patients P2 and P3 and other published cases with *SECISBP2* defects (P5[1], P6[2], P7[2]), with β-Actin denoting comparable protein loading**.** Image of one experiment.

D. Heat map of relative mRNA levels of selenoproteins and *SECISBP2* in fibroblasts from control subjects (C) and patient P3 measured by qPCR. Red indicates higher and blue lower levels of gene expression relative to the control (uniformly designated yellow). Three independent experiments.

E. Thioredoxin reductase activity in dermal fibroblasts from P3 compared to controls (C1, C2, C3). Three independent experiments.

F. H_2O_2 production in dermal fibroblasts from P3 compared to controls (C1, C2, C3). n=14 independent experiments.

E-F. Statistics: ordinary one-way ANOVA with adjusted P values (Tukey's multiple comparison test), each bar represents the mean value, error bars represent SEM.

Source data are provided as a Source data file.

Supplementary Figure 4: *SECISBP2* genotype and biochemical phenotype in patient P4 and family members.

A

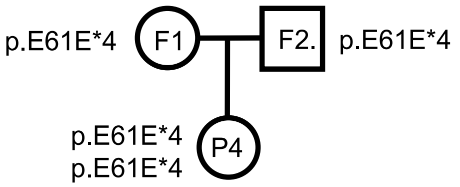

B

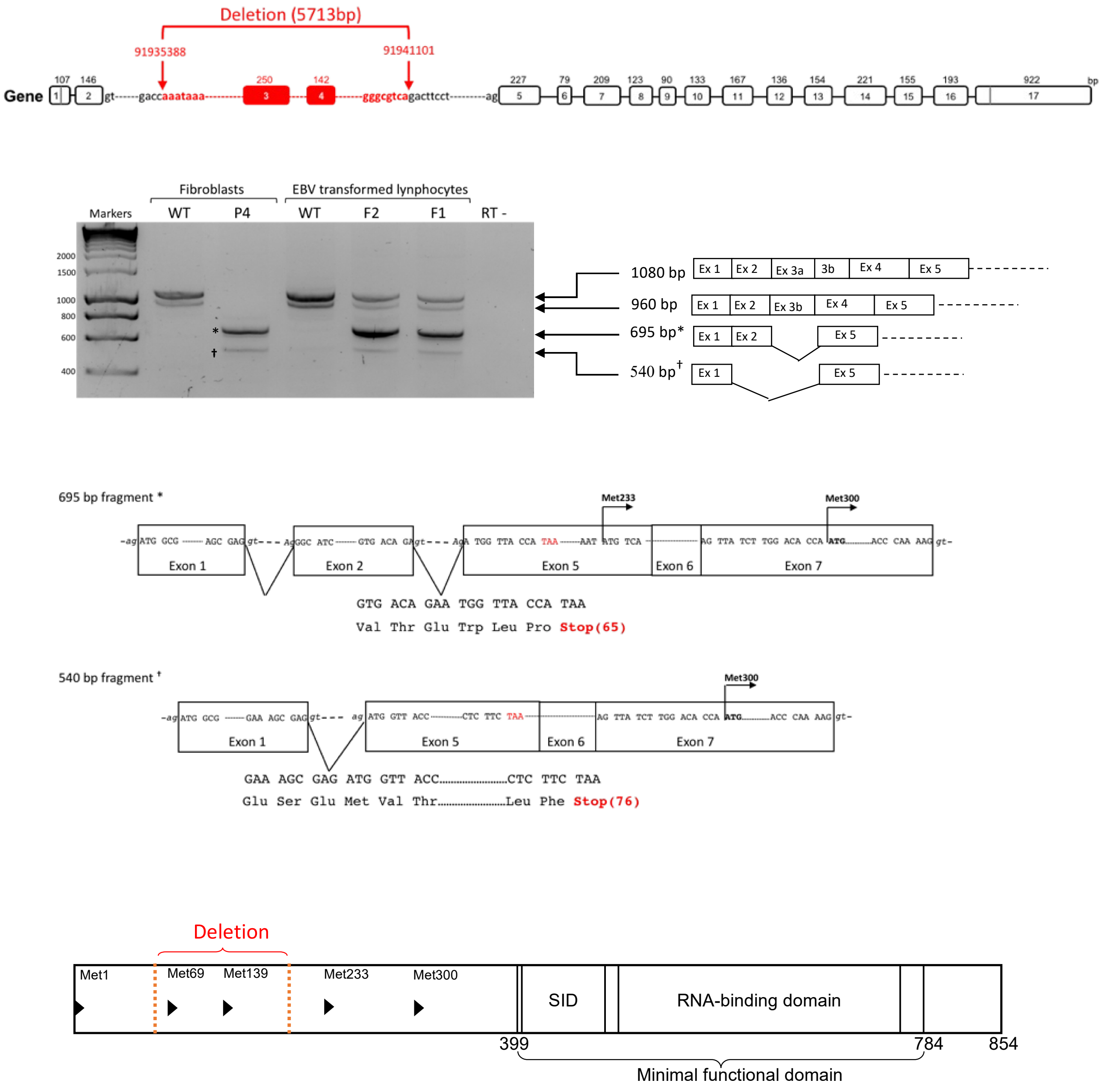

C

	P4	F1	F2
TSH (rr mU/L)	4.29 (0,35-5.5)	3.4 (0,35-5.5)	3.83 (0,35-5.5)
T4 (rr nmol/L)	204.9 (69-141)	130.7 (69-141)	126.6 (69-141)
FT4 (rr nmol/L)	37.6 (10.5 – 21.0)	15.3 (10.5 – 21.0)	18.3 (10.5 – 21.0)
FT3 (rr nmol/L)	3.6 (4.2 – 7.6)	4.4 (3.5 – 6.5)	4.9 (3.5 – 6.5)
rT3 (rr nmol/L)	0.74 (0.12 – 0.36)	0.12 (0.12 – 0.36)	0.20 (0.12 – 0.36)
Selenium (rr mmol/L)	0.3 (0.70 – 1.5)	1.21 (0.90 – 1.7)	1.25 (0.90 – 1.7)

A. Family pedigree and *SECISBP2* genotype of patient P4 and her parents (F1, F2), with squares and circles denoting male and female individuals respectively.

B. Schematic representation of coding exons of *SECISBP2*, with the location of a 5713bp deletion (Chr9:91935388-91941101del) identified in P4, which encompasses exons 3 and 4, superimposed (top).

Aberrantly spliced SECISBP2 transcripts in P4 and family members. RT-PCR (RT-: negative control; M: DNA molecular weight markers) of dermal fibroblast (P4) or EBV-transformed lymphocyte RNA from parents (F1, F2) and controls (WT), using forward (exon 1) and reverse (exon 7) primers, generates different products. Two ubiquitous bands (1080bp and 960bp), corresponding to known, natural SECISBP2 splice variants are identified in WT, F1 and F2; only aberrantly spliced variants (695bp (*) and 540bp (†) were found in P4 or heterozygously in each parent (middle).

Translation of aberrantly spliced variant (* and †) transcripts, predicts premature termination of translation at stop codons (65,76) in exon 5 (red) (lower).

ATG codons (triangles) in exon 5 (Met233) or exon 7 (Met300) downstream of this, which could be sites for reinitiation of SECISBP2 synthesis, encompassing Sec incorporation (SID) and RNA-binding domains of the minimal functional protein, are indicated (bottom).

C. Circulating thyroid hormones and biochemistry (TSH, thyroid stimulating hormone; T4, total thyroxine; FT4, free thyroxine; T3, total triiodothyronine; rT3, reverse triiodothyronine) in patient P4 and family members (F1-2). High values are shaded red and low values blue.

Supplementary Figure 5: Selenoprotein deficiency in patient P4.

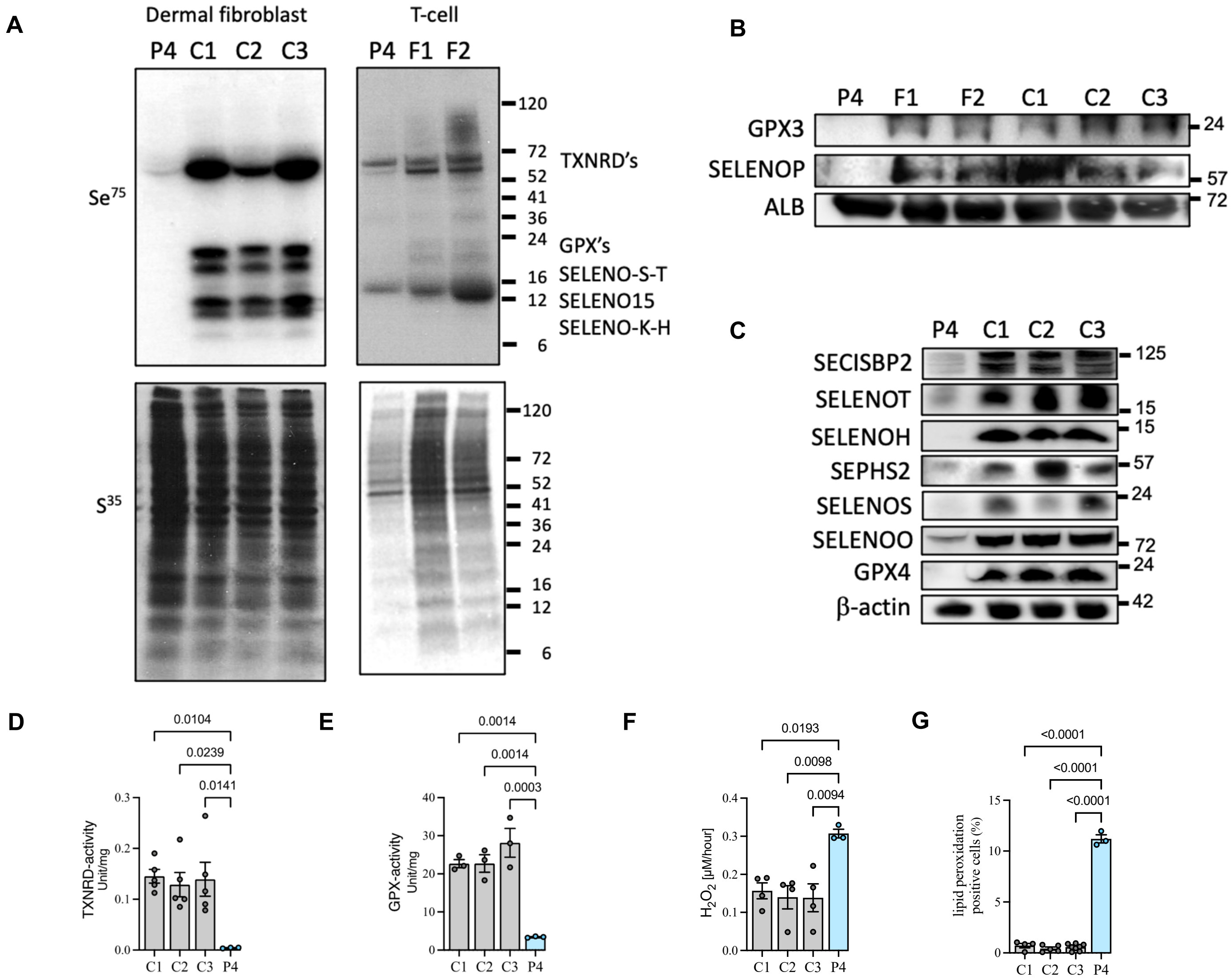

A. ^{75}Se labelling of primary dermal fibroblast and T-cells from patient P4 compared to three unrelated controls (C1, C2, C3) parents (F1, F2) (upper panel). ^{35}S-Met labelling confirmed comparable protein loading (lower panel). Image of one experiment.

B. Western blot of serum selenoproteins (GPX3, SELENOP) from patient P4, parents (F1, F2) and three unrelated controls (C1, C2, C3), with albumin (ALB) as loading control. Representative image of two independent experiments.

C. Western blot of selenoproteins in dermal fibroblasts from patient P4 and controls (C1, C2, C3) with β-Actin as loading control**.** Image of one experiment.

D. Thioredoxin reductase activity in dermal fibroblasts from P4 compared to controls (C1, C2, C3).

E. Glutathione peroxidase activity in dermal fibroblasts from P4 compared to controls (C1, C2, C3).

F. H_2O_2 production in dermal fibroblasts from P4 compared to controls (C1, C2, C3).

G. Lipid peroxidation in dermal fibroblasts from P4 compared to controls (C1, C2, C3).

D-G. Three independent experiments. Statistics: ordinary one-way ANOVA with adjusted P values (Tukey's multiple comparison test), each bar represents the mean value, error bars represent SEM.

Source data are provided as a Source data file.

Supplementary Figure 6: Membrane lipid peroxidation and apoptosis of aortic vascular smooth muscle cells.

A

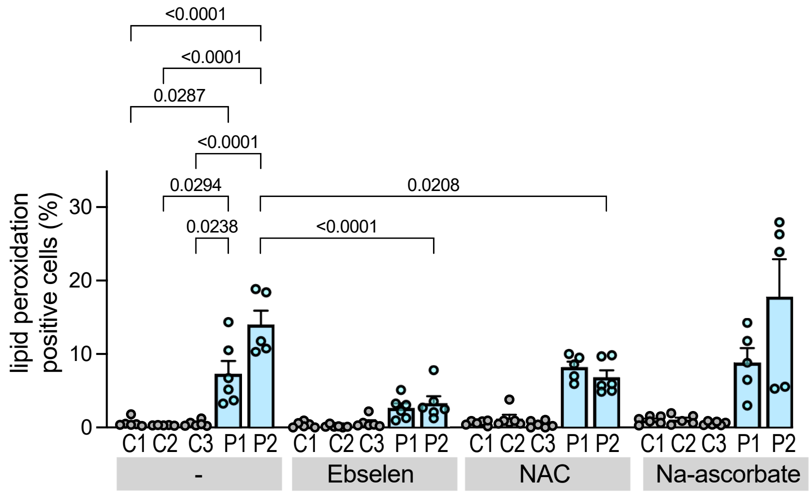

B

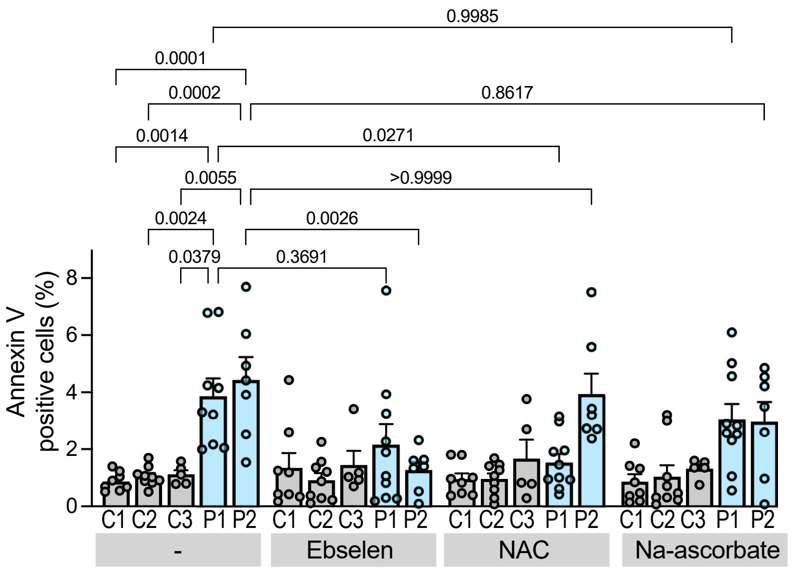

A-B. membrane lipid peroxidation (**A**) and apoptosis (Annexin V positive cells) (**B)** of aortic VSMCs from controls (C1, C2, C3) and patients P1, P2 following exposure to ebselen, N-acetylcysteine (NAC) or Na-L-ascorbate. n=5-10 independent experiments.

Statistics: Two-way ANOVA with adjusted P values (Tukey's multiple comparison test), each bar represents the mean value, error bars represent SEM. Source data are provided as a Source data file.

Supplementary Figure 7: Selenoprotein deficiency in *Secisbp*$^{Q333X/Q333X}$ mutant zebrafish.

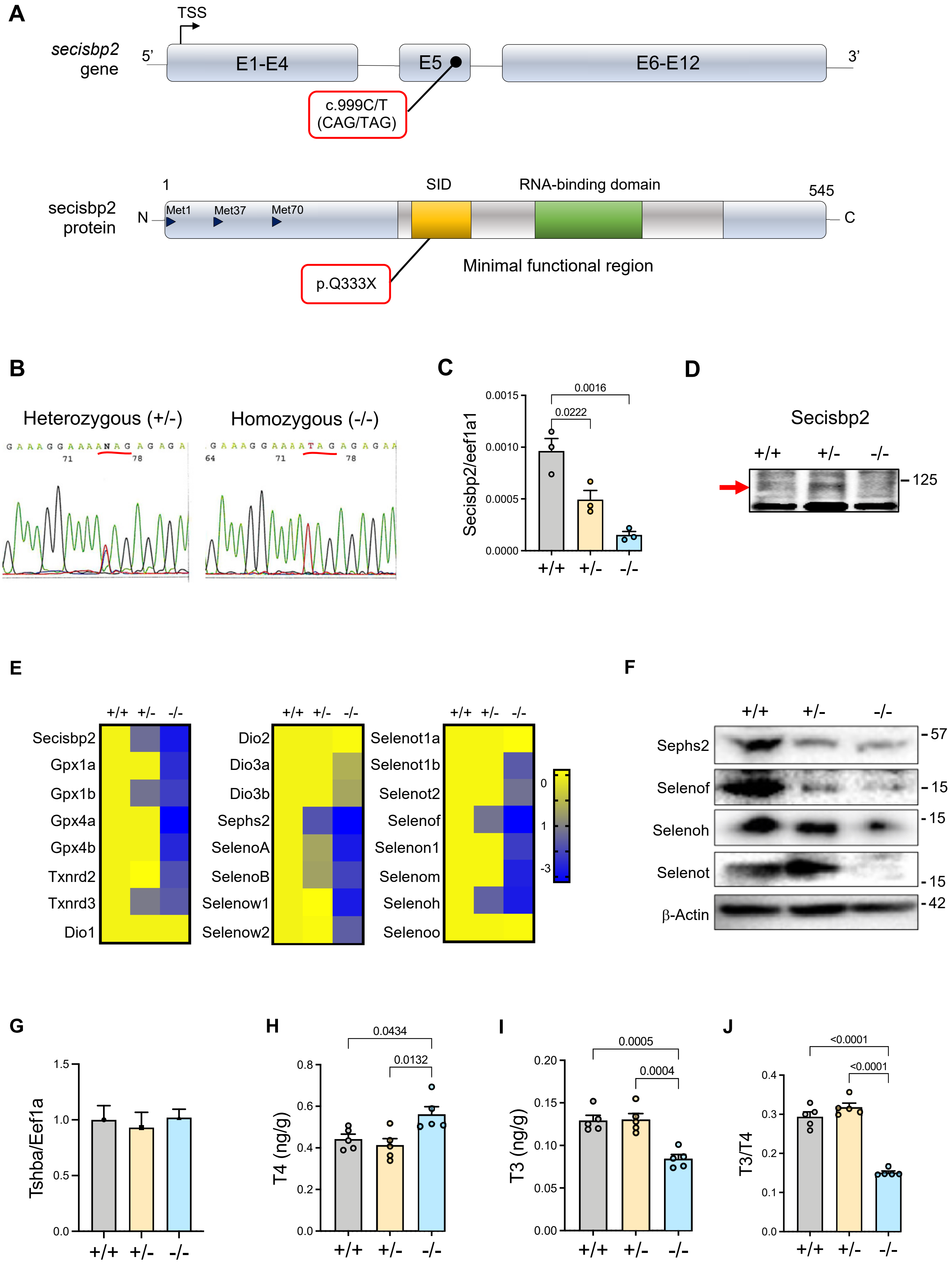

A. Schematic of genomic organisation (top) and protein structure (bottom) of zebrafish Secisbp2, depicting the functional domains and the position of the Q333X mutation. The nucleotide change (CAG/TAG) in exon 5 corresponds to a stop mutation (p.Q333X), which prematurely truncates the minimal functional region (grey), encompasses the Sec incorporation domain (SID) and RNA binding domain.

B. Electropherograms showing the heterozygous (left) and homozygous (right) C/T nucleotide substitutions in *secisbp2* mutant zebrafish.

C. *Secisbp2* mRNA expression in wild type (+/+), heterozygous (+/-) and homozygous (-/-) zebrafish embryos at two days post-fertilization (n = 40 embryos). Two independent experiments.

D. Western blot of Secisbp2 protein in wild-type (+/+), heterozygous (+/-) and homozygous (-/-) embryos at two days post-fertilization (n = 50 embryos). Image from one experiment.

E. Heat map of selenoprotein and Secisbp2 mRNA expression in wild-type (+/+), heterozygous (+/-) and homozygous (-/-) embryos at two days post-fertilization (n = 40 embryos). Blue indicates lower levels of gene expression relative to the control (uniformly designated yellow). Three independent experiments.

F. Western blot of selenoproteins (Sephs2, Selenof, Selenoh, Selenot) or β-actin (loading control) in wild-type (+/+), heterozygous (+/-) and homozygous (-/-) embryos at four days post-fertilization (n = 50 embryos). Image from one experiment.

G-J. Quantitation of *tshba* mRNA (n=3). or thyroid hormone (thyroxine, T4; triiodothyronine) levels in adult (age 6 months, n=5) wild-type (+/+), heterozygous (+/-) or homozygous (-/-) mutant zebrafish. Three independent experiments.

Statistics: Ordinary one-way ANOVA with adjusted P values (Tukey's multiple comparison test), each bar represents the mean value, error bars represent SEM.

Source data are provided as a Source data file.

Supplementary Figure 8: Selenoprotein deficiency in Secisbp2 knockdown zebrafish.

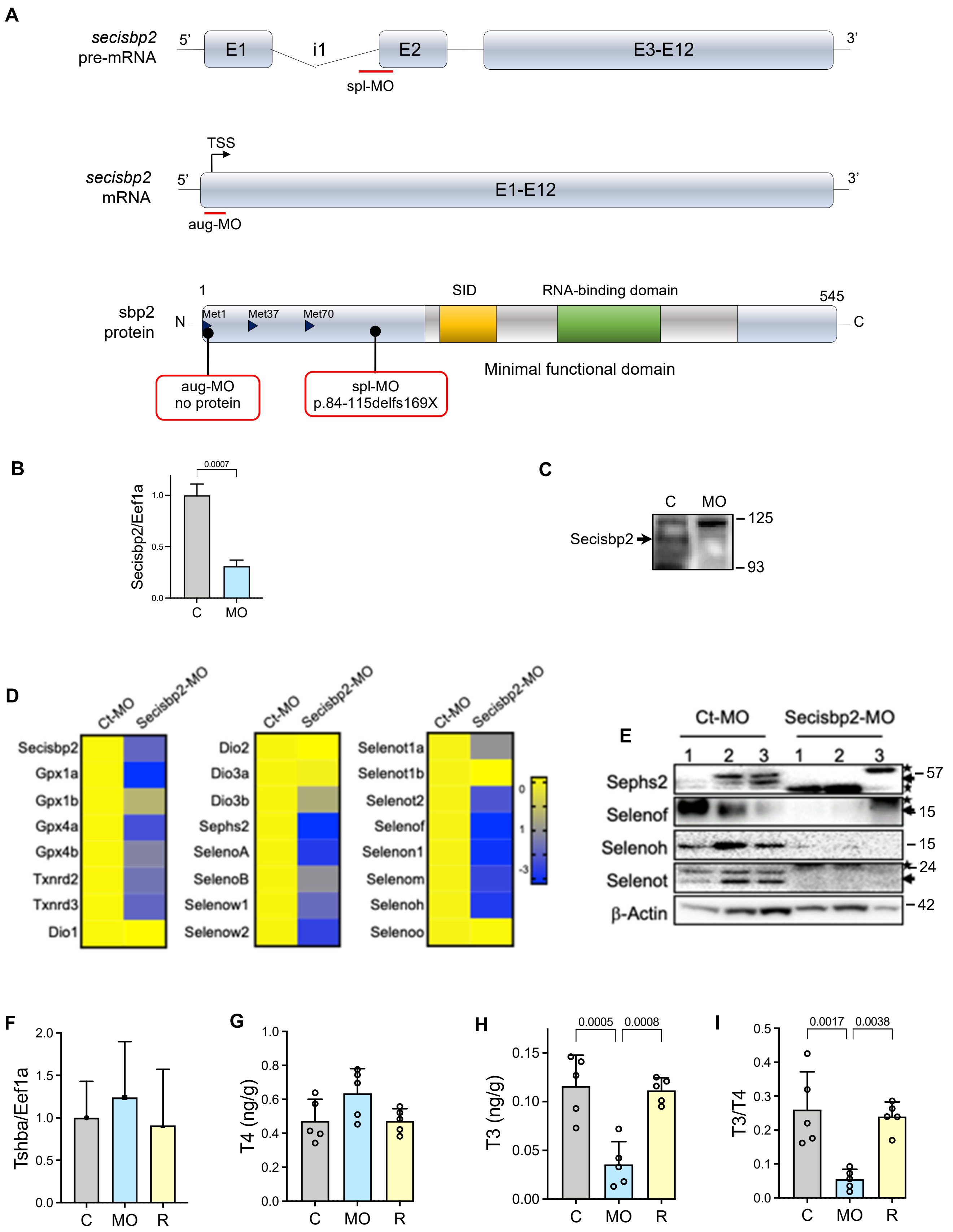

A. Schematic of zebrafish *secisbp2* gene (top) mRNA (middle) and protein (bottom), with the location of morpholinos targeting the intron 1 - exon 2 boundaries (i1-E2) (spl-MO) (top) and transcription start site (TSS) (aug-MO) superimposed. The spl-MO is pre1dicted to generate a mis-spliced transcript which truncates the protein prematurely (p.84-115delfs169X), with the aug-MO blocking synthesis from the first methionine codon (Met1) (www.predictprotein.org), corresponding to loss of the minimal functional region encompassing the Sec incorporation domain (SID) and RNA binding domain.

B. *Secisbp2* mRNA expression in embryos at two days post fertilization following injection of control (C) or *secisbp2* (MO) morpholinos. Statistics: Unpaired t test, each bar represents the mean of three independent experiments with 40 pooled embryos, error bars represent SD.

C. Western blot of Secisbp2 protein in embryos at two days post fertilization following injection of control (Ct-MO) or *secisbp2* (Secisbp2-MO) morpholinos (n= 50 embryos). Representative image of two independent experiments.

D. Heat map of selenoprotein and Secisbp2 mRNA expression in embryos at two days post fertilization following injection of control (Ct-MO) or *secisbp2* (Secisbp2-MO) morpholinos. Blue indicates lower levels of gene expression relative to the control (uniformly designated yellow). Three independent experiments with 40 pooled embryos.

E. Western blot of selenoproteins (Sephs2, Selenof, Selenoh, Selenot) or β-actin (loading control) in embryos at four days post fertilization following injection of control (Ct-MO) or *secisbp2* (Secisbp2-MO) morpholinos. Arrows and stars denote specific and non-specific bands respectively. Image of one experiment witrh 50 pooled embryos.

F- I. Quantitation of *tshba* mRNA (n=3) or thyroid hormone (thyroxine, T4; triiodothyronine) levels in embryos at four days post fertilization following injection of control (Ct-MO) or *secisbp2* (Secisbp2-MO) morpholinos (n= 50 embryos). Five independent experiments with 40 pooled embryos.

Statistics: Ordinary one-way ANOVA with adjusted P values (Tukey's multiple comparison test), each bar represents the mean value, error bars represent SEM. Source data are provided as a Source data file.

Supplementary Figure 9: Mouse model with conditional inactivation of *Secisbp2* in aortic VSMCs.

A

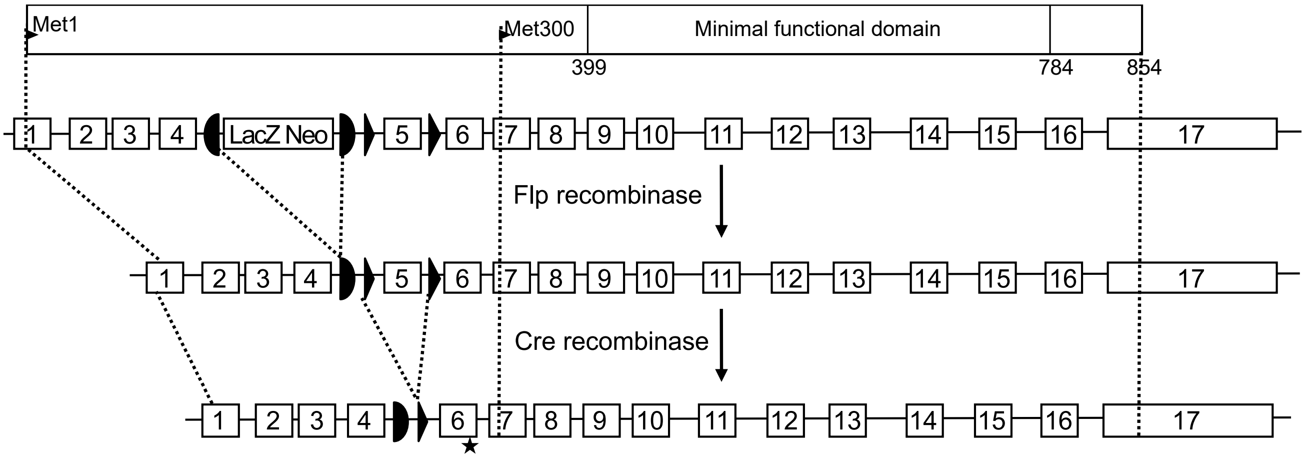

B

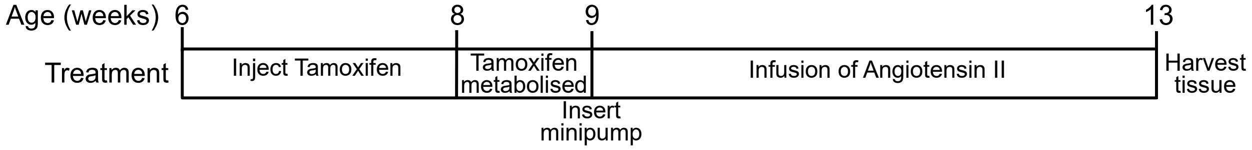

C

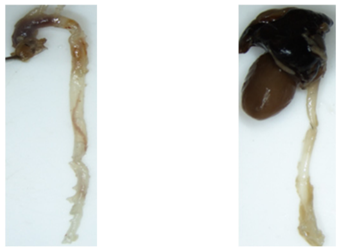

A. Schematic showing structure of the targeted *Secisbp2* allele before and after deletion events, aligned with full-length Secisbp2 protein. Flp-FRT recombination achieved by germline expression of FLP recombinase removes the gene-trap cassette containing an En2 splice acceptor, in-frame lacZ/neo reporter/selection cassette and polyadenylation signal (LacZ-Neo), leaving a conditional (floxed) exon 5. Cre-mediated recombination using male, Myh11-Cre^{ERt2} mice with VSMC specific Cre-expression removes exon 5 of *Secisbp2,* generating a frameshift and premature stop (asterisk) in exon 6 resulting in Myh11-CreERt2/Secisbp2$^{flox/flox}$ animals. Flippase recognition target (FRT) sites are denoted as half circles and locus of X-over P1 (loxP) sites as triangles.

B. Experimental protocol for the mouse studies. Myh11-Cre^{ERt2}/Secisbp2$^{WT/WT}$, Myh11-Cre^{ERt2}/Secisbp2$^{WT/flox}$ and Myh11-Cre^{ERt2}/Secisbp2$^{flox/flox}$ male animals were injected with 10 x 1 mg tamoxifen from week 6 to 8, followed by a rest period of 1 week before insertion of a minipump for Angiotensin II infusion. After 28 days of Angiotensin II infusion the aorta was harvested for further analysis.

C. Representative macroscopic images of the aortic tree from Myh11-Cre^{ERt2}/Secisbp2$^{flox/flox}$ male animals following the experimental protocol described in **B**. Representative image of three independent experiments.

Supplementary Figure 10: Genotyping mice to confirm conditional inactivation of *Secisbp2* in vascular smooth muscle cells.

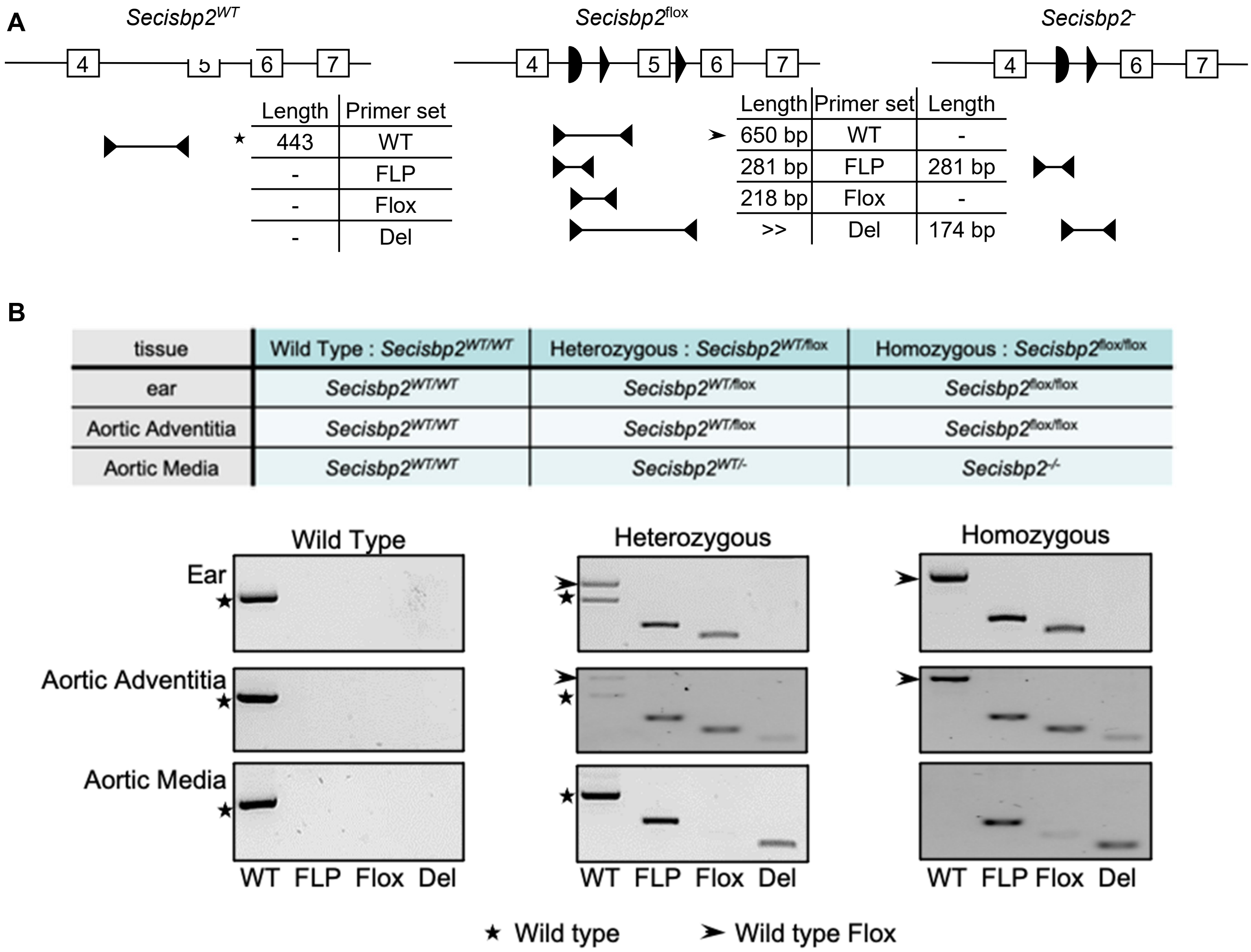

A. Schematic depicting the genotyping strategy used to detect the wild type *Secisbp2* allele and the targeted alleles before (*Secisbp2*^{flox}) and after Cre-mediated recombination (*Secisbp2*$^{-}$). Four primer sets were used: WT: detects the wild-type allele (asterixed 443bp product), and generates a larger amplicon (arrowhead, 650 bp) for *Secisbp2*^{flox}; FLP: detects the recombined FRT-site (half circle) present in the targeted alleles (*Secisbp*^{flox} and *Secisbp2*$^{-}$); Flox: detects the floxed exon 5 (only present in *Secisbp2*^{flox}); Del: detects deletion of exon 5 (*Secisbp2*$^{-}$ only).

B. Top: Table showing expected genotypes with analyses of genomic DNA from ear, aortic adventitia and aortic media tissue following tamoxifen-injection of Myh11-Cre^{ERt2}-positive animals that are wild type (*Secisbp2*$^{WT/WT}$), heterozygous (*Secisbp2*$^{WT/flox}$) and homozygous (*Secisbp2*$^{flox/flox}$).

Bottom: Representative genotyping results of tamoxifen-injected Myh11-Cre^{ERt2} positive animals. The deleted (Del) allele of *Secisbp2* is only detected in the aortic media, due to recombination in vascular smooth muscle cells, where Cre is specifically expressed. Low intensity bands for Flox in aortic media and Del in aortic adventitia reflect low level contamination after manual separation of the cell layers.

Supplementary Figure 11: Immunohistochemical analyses of macrophage markers in human or murine aortas

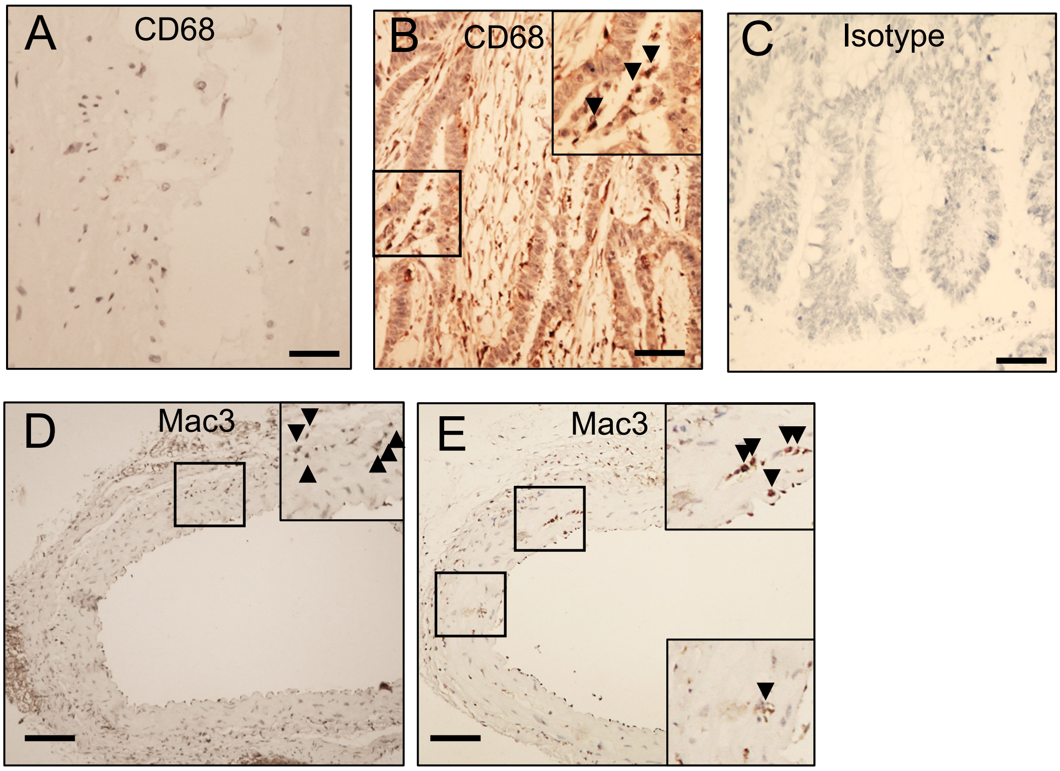

A. Histology of region of aortic degeneration from patient P1b stained with antibody to CD68.

B-C. Positive control tissue (colon cancer) stained with anti-CD68 (**B**) or isotype control antibody (**C**).

D-E. Histology of male mouse thoracic aorta stained with antibody to MAC-3 in wild type control (**D**) or VSMC-specific *Secisbp2* knockout (**E**) mice. Insets show high power views of outlined regions, arrowheads indicate positive cells. Scale bars are 25μm in A, 100μm in B-C, 200μm in D,E.

A-E. n=1 experiment.

Supplementary Figure 12: Example of flow cytometry gating strategy.

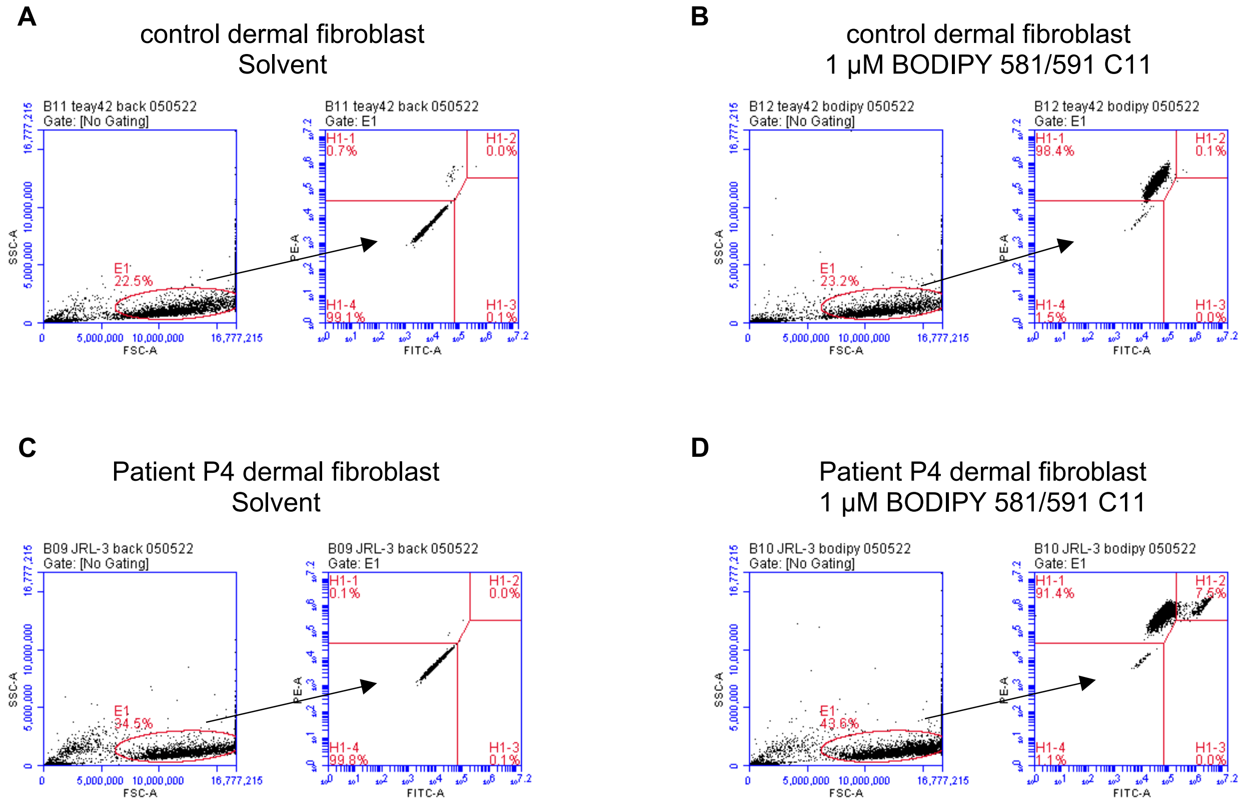

A-D. Dermal fibroblast cells from controls (**A, B**) and patient P4 (**C, D**) treated with solvent (methanol, (**A,C**)) or Bodipy (**B,D**). Gating was applied to select the main healthy cell population. After treatment with bodipy, FL2 shift (585nm, PE) indicates uptake of the dye and the FL1-shift (FITC, 530nm) indicates for membrane lipid peroxidation. The cells in the H1-2 quadrant indicate the percentage of cells positive for membrane lipid peroxidation.

Supplementary Figure 13: Expression of vascular smooth muscle cell-specific genes and protein products in aortic vascular smooth muscle cells.

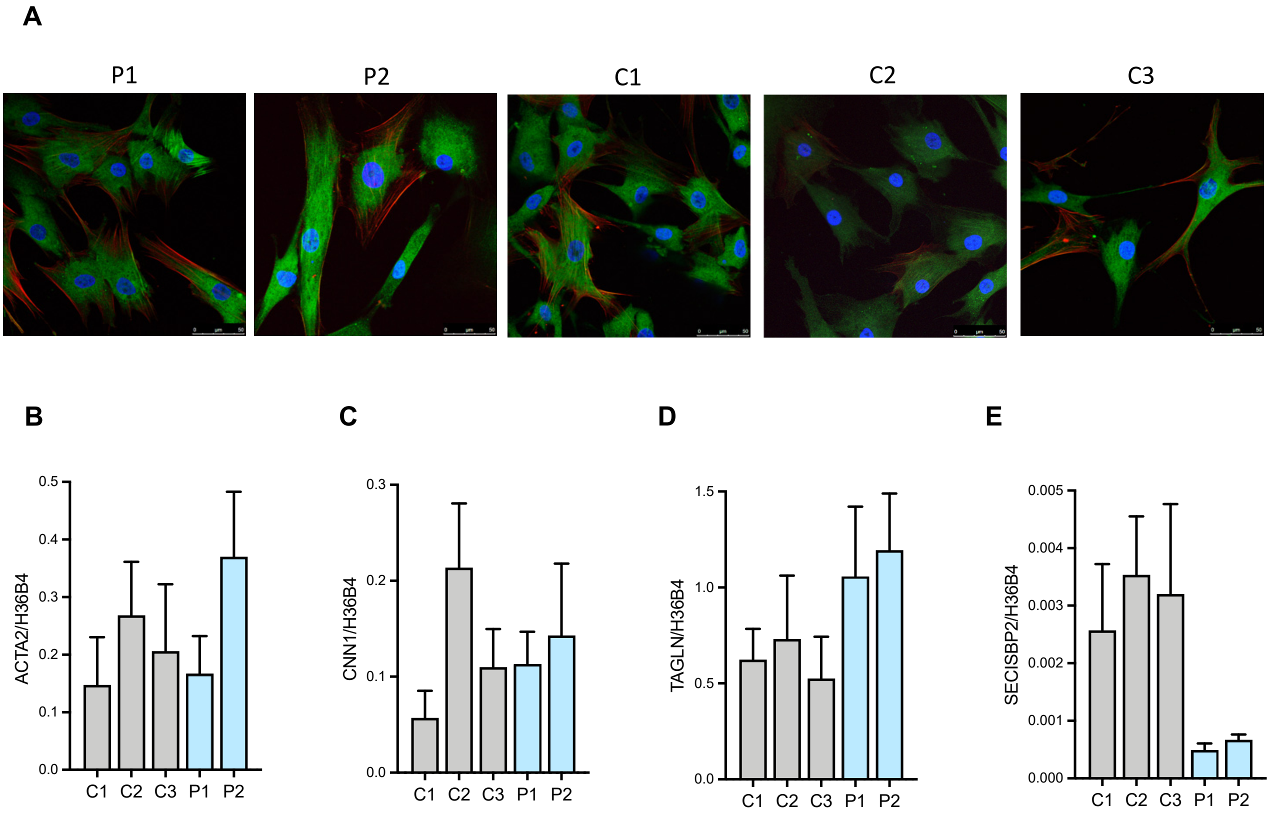

A. Representative confocal images of immunofluorescence performed on VSMC from patient P1 and P2 and controls (C1, C2, C3) using ACTA2 (red) and CNN1 (green) antibodies (n=2 independent experiments).

B-E. ACTA2 (**B**), CNN1 (**C**), TAGLN (**D**) and SECISBP2 (**E**) mRNA expression in VSMC from patient P1 and P2 and controls (C1, C2, C3) (n= 3-10 independent experiments).

Statistics: Ordinary one-way ANOVA with adjusted P values (Tukey's multiple comparison test; no significant differences), each bar represents the mean value, error bars represent SEM.

Source data are provided as a Source data file.

Supplementary Tables

Supplementary Table 1: Aortic traits in carriers of heterozygous, loss-of-function (LOF), *SECISBP2* variants in UK Biobank

Trait	Number of samples (1)	cMAF (2)	No of LOF carriers	Effect size (3)	SE(4)	P-value
Ascending Aorta Diameter in diastole	34618	0.000332197	23	-0.06367	0.096	0.510
Ascending Aorta Diameter in diastole (IRN) (5)	34618	0.000332197	23	-0.15454	0.175	0.378
Ascending Aorta Diameter in systole	34618	0.000332197	23	-0.08533	0.099	0.392
Ascending Aorta Diameter in systole (IRN)	34618	0.000332197	23	-0.21487	0.177	0.226
Descending Aorta Diameter in diastole	35381	0.000353297	25	-0.03278	0.056	0.562
Descending Aorta Diameter in diastole (IRN)	35381	0.000353297	25	-0.20047	0.154	0.195
Descending Aorta Diameter in systole	35381	0.000353297	25	-0.02819	0.059	0.636
Descending Aorta Diameter in systole (IRN)	35381	0.000353297	25	-0.18298	0.154	0.236

cMAF cutoff: 0.1%; (1) number of samples with MRI and exome samples available; (2) cumulative minor allele frequency; (3) in cm for raw traits or in number of standard deviations for inverse-rank-normalized (IRN) traits; (4) standard error of the effect size; (5) inverse-rank normalized

Supplementary Table 2: Oligonucleotides for analysis of misspliced SECISBP2 transcript in patient P1

DNA primers to detect splice variants	
**Name**	**Sequence**
rx2-F	AAGCAGCGTCTCCAAGAAAA
rx2-R	CTGAAGATCCCCACCACACT

Supplementary Table 3: Antibodies for western blotting of human, zebrafish and mouse selenoproteins

Protein	Catalogue No	Manufacturer	dilution
GPX1	ab108427	Abcam	1/1000
GPX3	SC58361	Santa Cruz	1/1000
GPX4	sc50497	Santa Cruz	1/1000
SEPHS2	ab153878	Abcam	1/500
βActin	ab8227	Abcam	1/2000
SECISBP2	EB07428	Everest Biotech	1/500
SELENOO	ab172957	Abcam	1/1000
SELENOS	HPA010025	Sigma	1/1000
SELENOH	ab151023	Abcam	1/1000
SELENOT	ab176192	Abcam	1/1000
SELENOI	H00085465-A01	abnova	1/1000
SELENOP	LF-MAO141	Ab Frontier	1/1000
SELENON	Gift from P Guicheney (INSERM, UMR S956, Pitié-Salpêtrière Hospital, Paris, France)		
SELENOF	ab124840	Abcam	1/1000
Albumin	Ab28405	Abcam	1/2000
Anti-rabbit IgG	7074	cell signaling	1/5000
anti-Mouse IgG	31430	invitrogen	1/2000
Anti-Goat/Sheep IgG	A9452	Sigma-Aldrich	1/5000

Supplementary Table 4: Oligonucleotides used in zebrafish studies

Application	Name	Sequence
Sequencing	Fw<br>Rw	GTCGTCCAGAAAGAGCCTTC<br>TGAACCGAACCATGAGACCA
Morpholino	aug-MO	CACAATGTTTATTCTGCCAGCTCAT
	spl-MO	GCGTGTTTGCACATCTTCACCTGCT
	Ct-MO	CCTCTTACCTCAGTTACAATTTATA
Quantitative PCR	*eef1a*-Fw<br>*eef1a*-Rw	CTGGTGTCCTCAAGCCTGGTA<br>ACTTGACCTCAGTGGTTACATTGG
	secisbp2-Fw<br>*secisbp2*-Rw	GTGGTCTAGATGAGGCTCTT<br>ACCTGTGCGCCATCATAGTT
	gpx1a-Fw<br>*gpx1a*-Rw	ACCCTGTGTCCCTTATGG<br>TGCTGTACCTCTTGAATGG
	gpx1b-Fw<br>*gpx1b*-Rw	CAACCAGTTCGGCTATCAGG<br>AACGGCATTGGCTCATCG
	gpx4a-Fw<br>*gpx4a*-Rw	AAACGTTGCCTCCAAATGAG<br>ATGACTTGGCGAATTCCTTG
	gpx4b-Fw<br>*gpx4b*-Rw	GCAGCAAGAGCTTCGCCAGAGCAAT<br>CTTCACCTGCAGACGTCCAGCGTTT
	txnrd2-Fw<br>*txnrd 2*-Rw	AACAAGCGTGGCAAAGAG<br>CAGTAGGGCAAAGAGAACAG
	txnrd 3-Fw<br>*txnrd 3*-Rw	TCAATGAGAAGAACGGGAAGGT<br>GCATAGATGTGAGGAACGTTGGT
	dio1-Fw<br>*dio1*-Rw	CGGACCCTGCTCAAAGAAGA<br>TCCGATGCCTCCCTGATAGA
	dio2-Fw<br>*dio2*-Rw	GCGTGCGCTCCATATGG<br>TTCGCCCAATTTCACCTGTT
	dio3a-Fw<br>*dio3a*-Rw	TCGCACCTGTATTCTCCGTG<br>CGAGCGTCCCGTATTCAGAC
	dio3b-Fw<br>*dio3b*-Rw	GCAGAGCGCATCCTGGATTA<br>CTCAGACGGGTCATGAAGGG
	sephs2-Fw<br>*sephs2*-Rw	GTGGTGCAGTTCGGGCTCTT<br>ACCTGTTTAGCATAATAGTT
	selenopa-Fw<br>*selenopa*-Rw	CAGGTGTGGCCGACTGACT<br>GGGTGGCTCAGGATGGTGTA
	selenopb-Fw<br>*selenopb*-Rw	ACCATCTGTCACTTCC<br>CTGCTCTTCCGGCTTTGC
	selenow1-Fw<br>*selenow1*-Rw	GCTGGTTGGAGGTGGAAGTA<br>TTGTTGCACTACCCAGGAGT
	selenow2-Fw<br>*selenow2*-Rw	CGGTTTCACAGACGCGGAAG<br>ACTCAGCAGTGACGACCCGC
	selenot1a-Fw<br>*selenot1a*-Rw	ACCCAGACATCCGCATAGAG<br>TGACTCCAAACCCAGATCCC
	selenot1b-Fw<br>*selenot1b*-Rw	GCGGAGTGTGCGGGATGGAA<br>ACTCTTCAAACACCCGCCGG
	selenot2-Fw<br>*selenot2*-Rw	TCAGGCCGACAAGATGAACT<br>AGATGCAGTCGATCCAGAGG
	selenof-Fw<br>*selenof*-Rw	CGTTATTACAGGGGTTGGCG<br>CTCCTGGGTAGAGCTTCCTG
	selenon1-Fw<br>*selenon1*-Rw	CACTGGCTACCTACCCAACA<br>CAAACTGCCCAGGAGTGAAC
	selenom-Fw<br>*selenom*-Rw	AACATCTGAGAACTGCCGGA<br>CATCATTAACGTCCCGCTGG
	selenoh-Fw<br>*selenoh*-Rw	GGTGTGTCAGATGTTCAGCC<br>TGTTCAACGCCTCTGATCCT
	selenoo-Fw<br>*selenoo*-Rw	GAACAGGGCAGGGTTACTCT<br>TTCACCAACACTTTCCGCAG
	tshba-Fw<br>*tshba*-Rw	AGTCTTCATGCTCATGGGAACAG<br>TGAACCCTATTAAAACCACACCT
	p53-Fw<br>p53-Rw	TGTAACTTGCACTTATTCACC<br>AGGCGCCAAGTTATCTCC
	casp3-Fw<br>casp3-Rw	GAACAGGAATGGGGGTTCGCA<br>GATCTTCCTGAGATGCTTTAT

Supplementary Table 5: Oligonucleotides for genotyping Secisbp2 mice

DNA primers for mouse genotyping	
**Name**	**Sequence**
Secisbp2_40098_F	TTTCGGTTGTTGCTGTTGTTG
Secisbp2_40098_R	AACTCCCCCTTTCCATCTGC
CAS_R1_Term	TCGTGGTATCGTTATGCGCC
Tm1c_F	AAGGCGCATAACGATACCAC
Tm1c_R	CCGCCTACTGCGACTATAGAGA
LR	ACTGATGGCGAGCTCAGACC
PhCREAS1	AGTCCCTCACATCCTCAGGTT
SMWT1	TGACCCCATCTCTTCACTCC
SMWT2	AACTCCACGACCACCTCATC

**Assay**	**Forward primer**	**Reverse primer**	**Product size (bp)**
Wild-type	Secisbp2_40098_F	Secisbp2_40098_R	650<br>443 (converted WT)
FLP	Secisbp2_40098_F	CAS_R1_Term	281
Flox	Tm1c_F	Tm1c_R	218
Del	Tm1c_F	LR	174

Supplementary Table 6: Quantitative PCR assays TaqMan assays

TaqMan Real-Time PCR Assays - Thermo Fisher Scientific	
**Gene**	**Catalogue no**
Mm01205647	Actb
Mm01354420	Secisbp2 ex5-6
Hs00218369	SELENOS
Hs00225349	SECISBP2
Hs00415057	SELENOH
Hs00892526	SELENOT
Hs01071062	SELENOW
Hs01013816	SELENOI
Hs01115694	SELENOM
Hs00188638	SELENOF
Hs00431229	SELENOK
Hs00972565	SELENOO

Supplementary Table 7: DNA primers for VSMC specific gene expression

DNA primers for VSMC specific gene expression[3]	
Name	Sequence
hACTA2-F	CACTGTCAGGAATCCTGTGA
hACTA2-R	CAAAGCCGGCCTTACAGA
hTAGLN-F	TCTTTGAAGGCAAAGACATGG
hTAGLN-R	TTATGCTCCTGCGCTTTCTT
hCNN1-F	GTCCACCCTCCTGGCTTT
hCNN1-R	AAACTTGTTGGTGCCCATCT

Supplementary Table 8: SECISBP2 patient var counts UKB exomes 394841

variant (chr-pos-ref-alt; build37)	variant (chr-pos-ref-alt; build38)	Patient	MAC	Homozygote counts	No non-missing alleles	MAF
9-91956271-A-T	9-89341356-A-T	P1	2	0	735566	2.71899E-06
9-91965544-C-G	9-89350629-C-G	P1	0	0	NA	0
9-91943668-91943668-DEL	9-89328753-89328753-DEL	P2	0	0	NA	0
9-91949282-T-A	9-89334367-T-A	P2	0	0	NA	0
9-91940541-C-T	9-89325626-C-T	P3	20	0	735918	2.72E-05
9-91935388-91941101del	9-89320473-89326186-DEL	P4	0	0	NA	0

Supplementary Table 9: SECISBP2 LOF variant counts UKBB MRI ascending aorta

Chromosome	Position	Reference allele	Alternative Allele	No non-missing genotypes	MAF	MAC	Consequence
9	89318600	GC	G	33603	1.49E-05	1	p.Glu10LysfsTer26
9	89319778	C	T	33616	1.49E-05	1	p.Gln55Ter
9	89319794	C	CA	33602	1.49E-05	1	p.Glu61ArgfsTer7
9	89325426	G	A	33593	1.49E-05	1	Splice acceptor
9	89325577	T	G	33615	1.49E-05	1	p.Tyr111Ter
9	89325601	C	A	33616	1.49E-05	1	p.Tyr119Ter
9	89325626	C	T	33616	1.49E-05	1	p.Arg128Ter
9	89325653	C	T	33618	1.49E-05	1	p.Gln137Ter
9	89325959	TG	T	33591	1.49E-05	1	p.166GlyfsTer
9	89325985	C	A	33614	1.49E-05	1	p.Ser174Ter
9	89328674	C	T	33564	1.49E-05	1	p.Arg197Ter
9	89339893	T	TG	33601	1.49E-05	1	p.Ala415GlyfsTer3
9	89339916	TAG	T	33594	1.49E-05	1	p.Glu423AspfsTer7
9	89341345	A	C	33556	1.49E-05	1	Splice acceptor
9	89341356	A	T	33586	1.49E-05	1	p.Lys438Ter
9	89341477	C	G	33616	1.49E-05	1	p.Ser478Ter
9	89346918	CAG	C	33616	2.97E-05	2	p.Gly495ProfsTer64
9	89348146	CCA	C	33549	1.49E-05	1	p.Ser558Ter
9	89348172	AGT	A	33609	1.49E-05	1	p.Ser566ArgfsTer3
9	89348215	G	C	33613	1.49E-05	1	Splice donor
9	89350635	C	A	33604	1.49E-05	1	p.Tyr632Ter
9	89358080	CTGGTGGGAGA	C	33612	1.49E-05	1	p.Leu784ArgfsTer26

Supplementary Table 10: SECISBP2 LOF variant counts UKBB MRI descending aorta

Chromosome	Position	Reference allele	Alternative Allele	No non-missing genotypes	MAF	MAC	Protein Consequence
9	89318600	GC	G	34343	1.46E-05	1	p.Glu10LysfsTer26
9	89319778	C	T	34356	1.46E-05	1	p.Gln55Ter
9	89319794	C	CA	34343	1.46E-05	1	p.Glu61ArgfsTer7
9	89325426	G	A	34334	1.46E-05	1	Splice acceptor
9	89325577	T	G	34356	1.46E-05	1	p.Tyr111Ter
9	89325601	C	A	34357	1.46E-05	1	p.Tyr119Ter
9	89325626	C	T	34357	2.91E-05	2	p.Arg128Ter
9	89325653	C	T	34359	1.46E-05	1	p.Gln137Ter
9	89325959	TG	T	34334	1.46E-05	1	p.166GlyfsTer
9	89325985	C	A	34355	1.46E-05	1	p.Ser174Ter
9	89328674	C	T	34305	1.46E-05	1	p.Arg197Ter
9	89339893	T	TG	34342	1.46E-05	1	p.Ala415GlyfsTer3
9	89339916	TAG	T	34334	1.46E-05	1	p.Glu423AspfsTer7
9	89341345	A	C	34297	1.46E-05	1	Splice acceptor
9	89341356	A	T	34327	1.46E-05	1	p.Lys438Ter
9	89341477	C	G	34357	1.46E-05	1	p.Ser478Ter
9	89346918	CAG	C	34357	4.37E-05	3	p.Gly495ProfsTer64
9	89348146	CCA	C	34289	1.46E-05	1	p.Ser558Ter
9	89348172	AGT	A	34348	1.46E-05	1	p.Ser566ArgfsTer3
9	89348215	G	C	34354	1.46E-05	1	Splice donor
9	89350635	C	A	34346	1.46E-05	1	p.Tyr632Ter
9	89358080	CTGGTGGGAGA	C	34353	1.46E-05	1	p.Leu784ArgfsTer26

Supplementary References

1. Schoenmakers, E., et al. Mutations in the selenocysteine insertion sequence–binding protein 2 gene lead to a multisystem selenoprotein deficiency disorder in humans. *J. Clin. Invest.* **120**, 4220-4235 (2010).

2. Dumitrescu, A. M., et al. Mutations in SECISBP2 result in abnormal thyroid hormone metabolism. *Nat. Genet.* **37**, 1247-1252 (2005).

3. Granata A., Serrano F., Bernard W.G., McNamara M., Low L., Sastry P., Sinha S. An iPSC-derived vascular model of Marfan syndrome identifies key mediators of smooth muscle cell death. *Nat Genet.* **49**, 97-109 (2017).

Uncropped scans of all blots

Supplementary Fig. 2: Selenoprotein deficiency in aortic vascular smooth muscle cells (VSMCs) from patients P1 and P2.

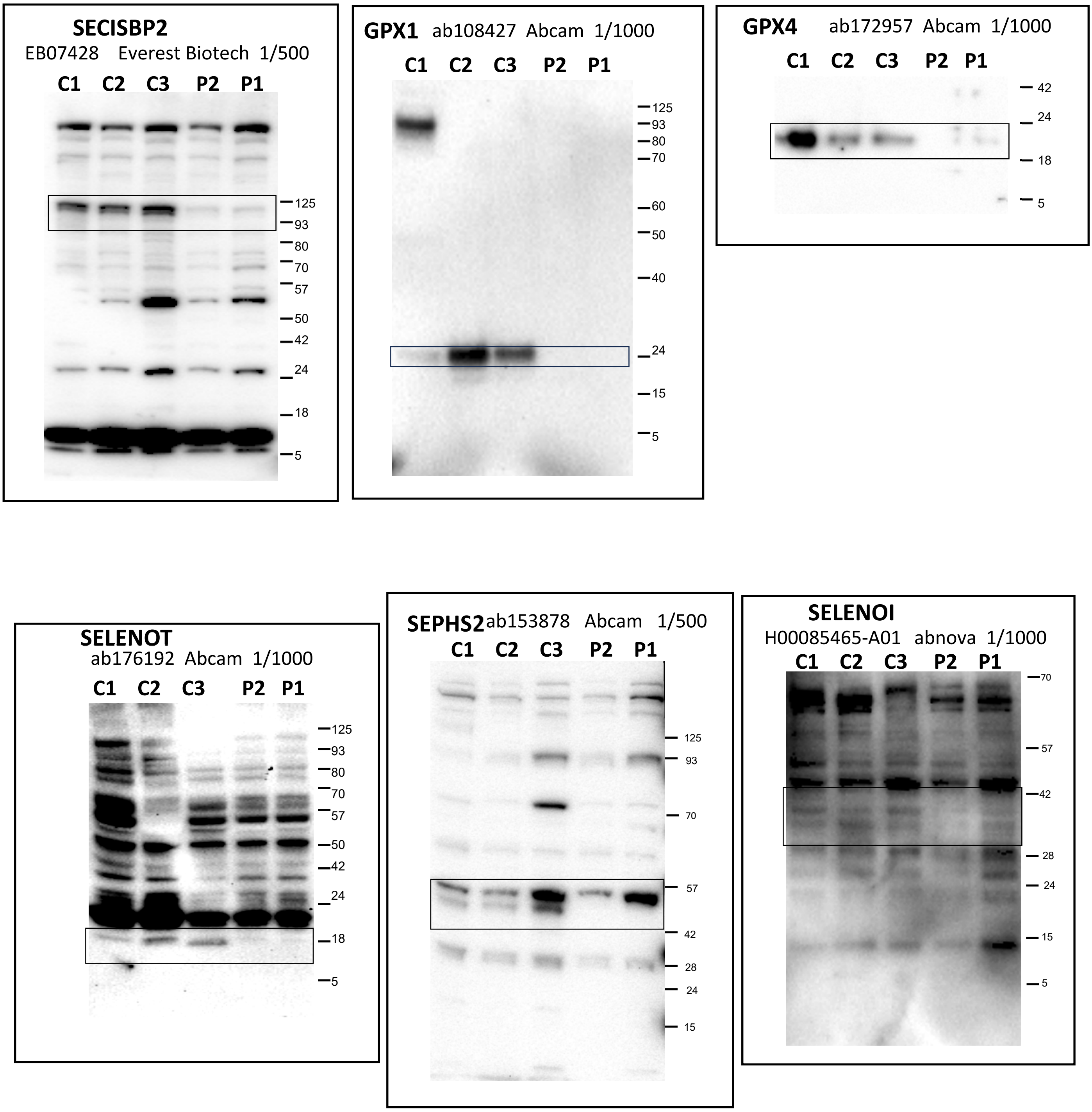

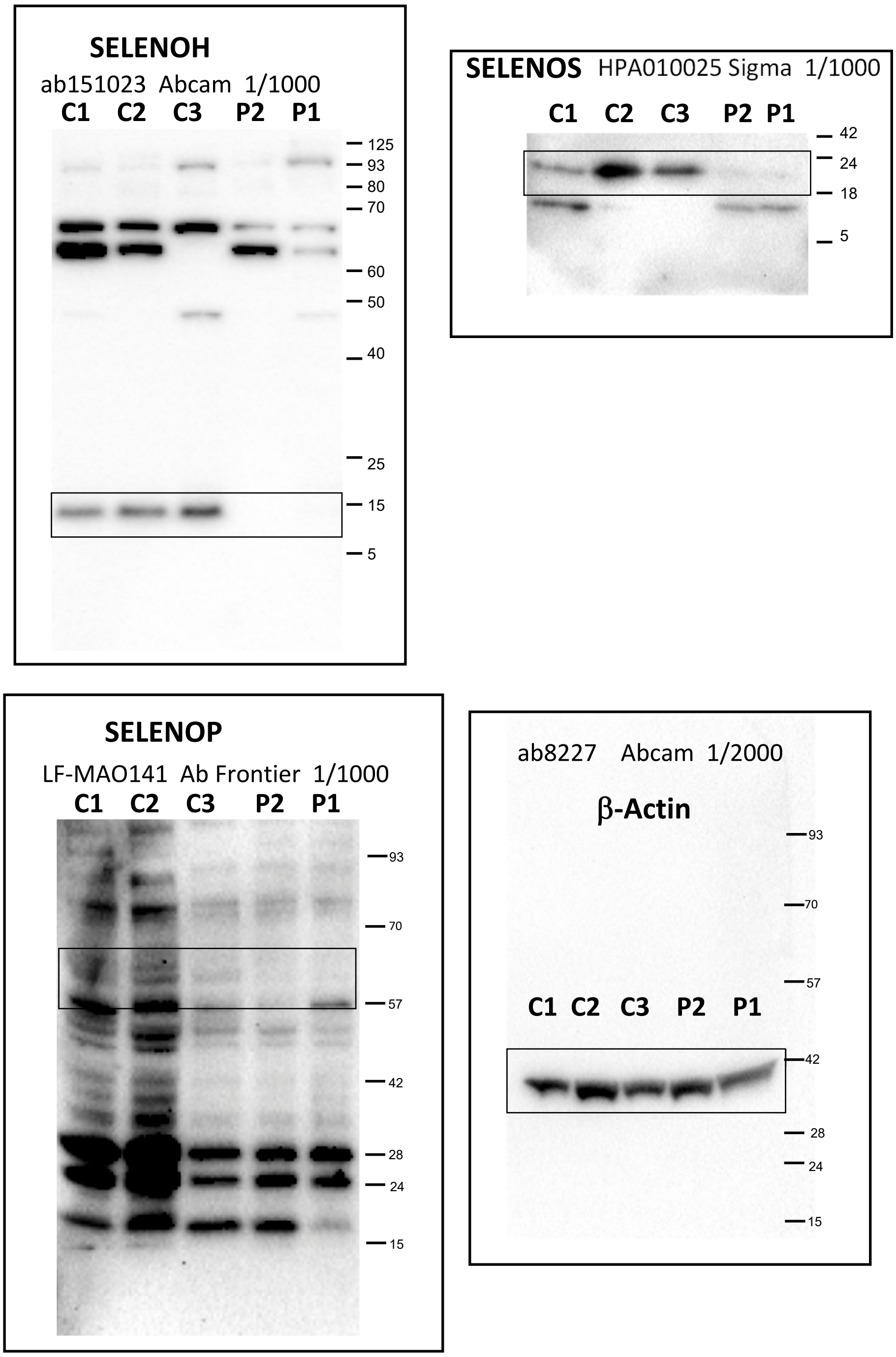
SELENOH
ab151023 Abcam 1/1000
C1 C2 C3 P2 P1
125
93
80
70
60
50
40
25
15
5
SELENOS HPA010025 Sigma 1/1000
C1 C2 C3 P2 P1
42
24
18
5
SELENOP
LF-MAO141 Ab Frontier 1/1000
C1 C2 C3 P2 P1
93
70
57
42
28
24
15
ab8227 Abcam 1/2000
β-Actin
93
70
57
C1 C2 C3 P2 P1
42
28
24
15

Supplementary Fig. 3B: Selenoprotein deficiency in patient P3.

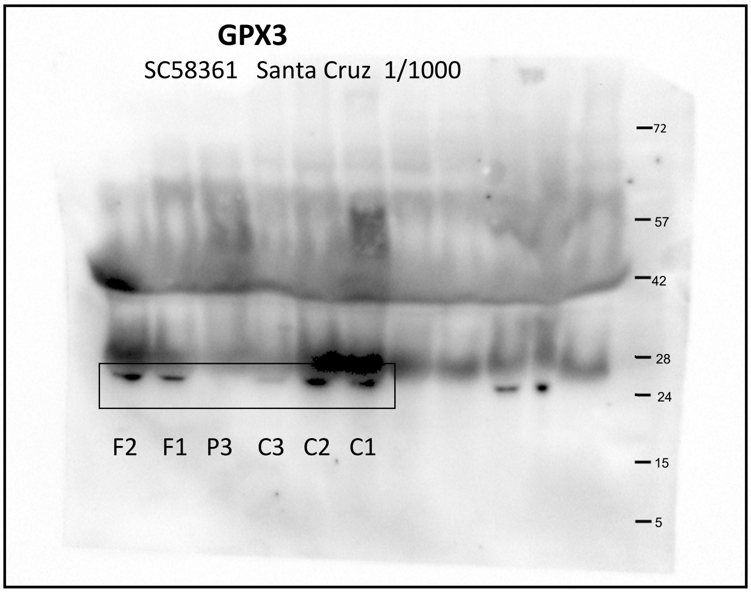

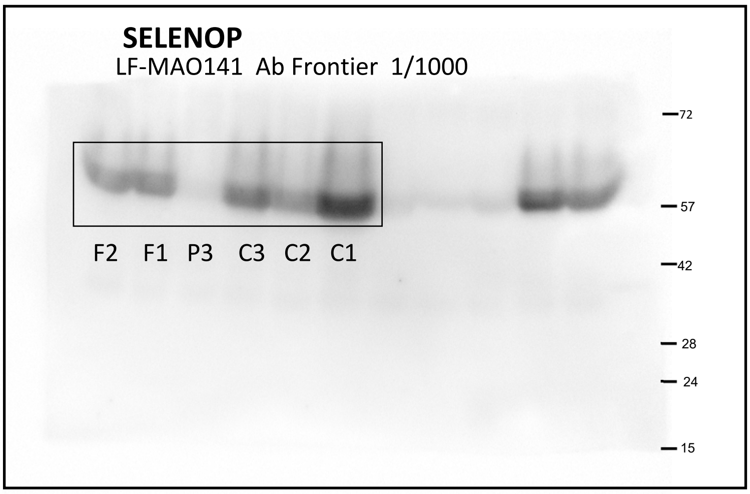

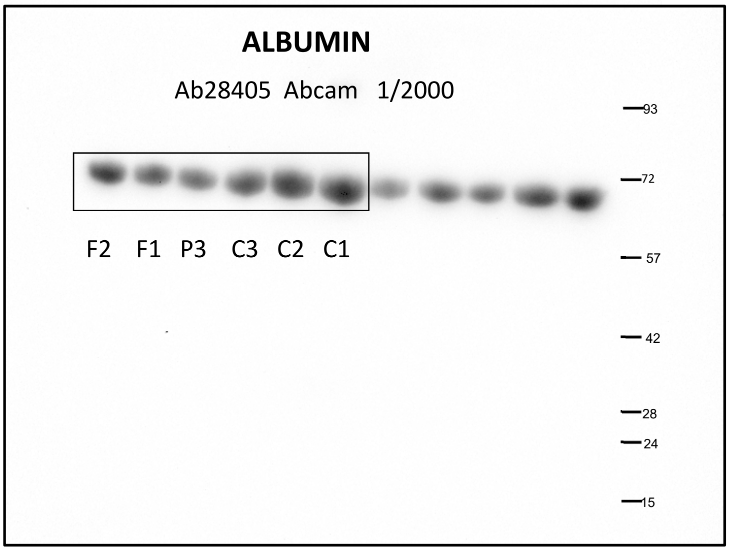

Supplementary Fig. 3C: Selenoprotein deficiency in patient P3.

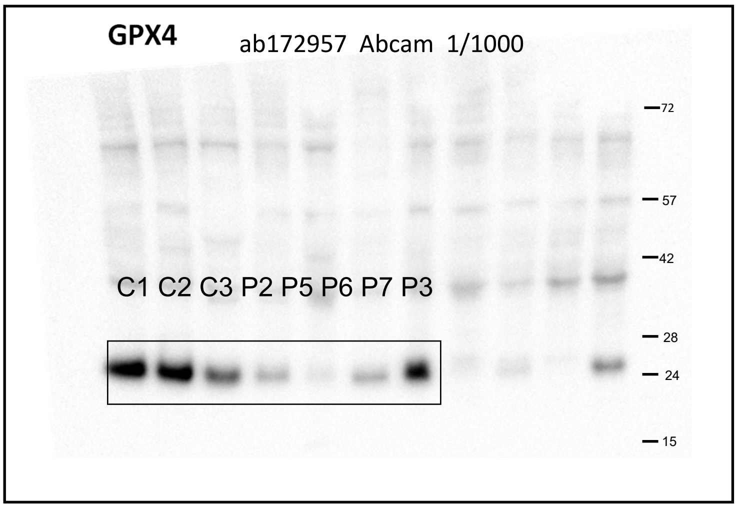

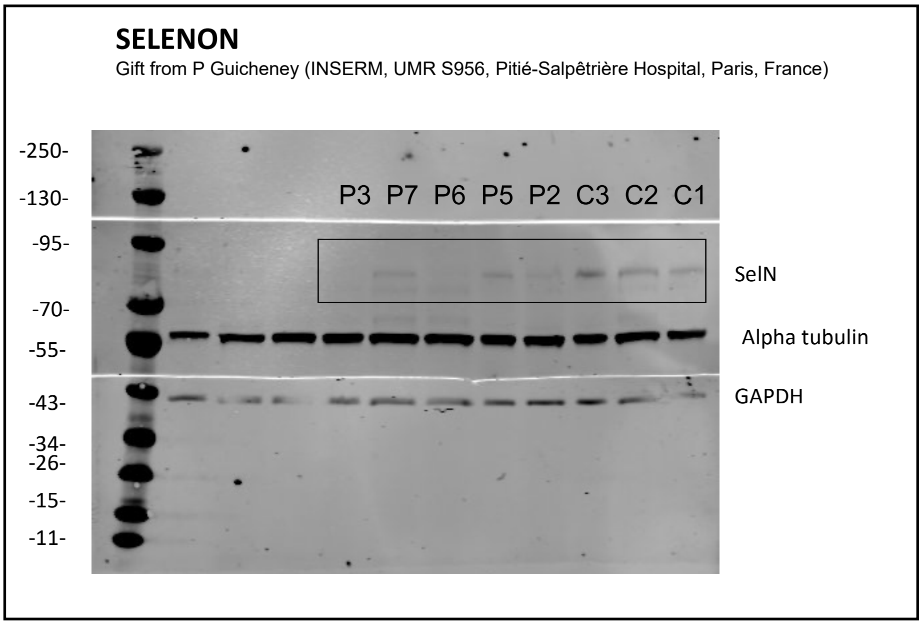

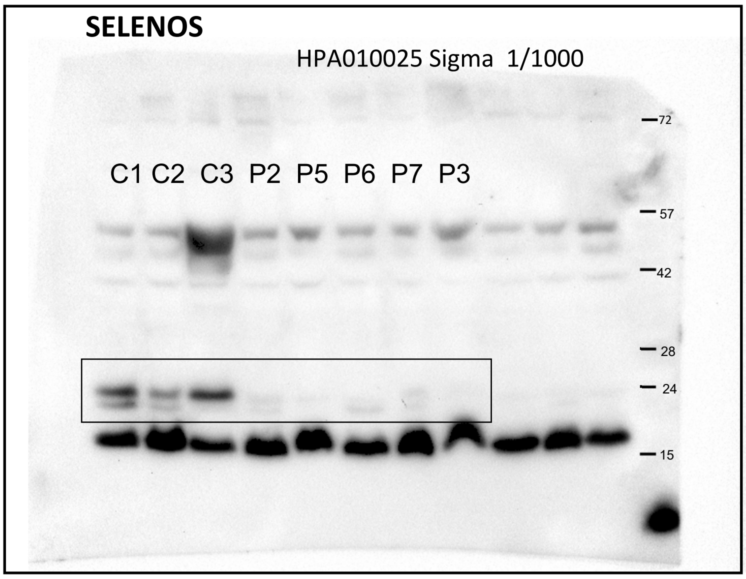

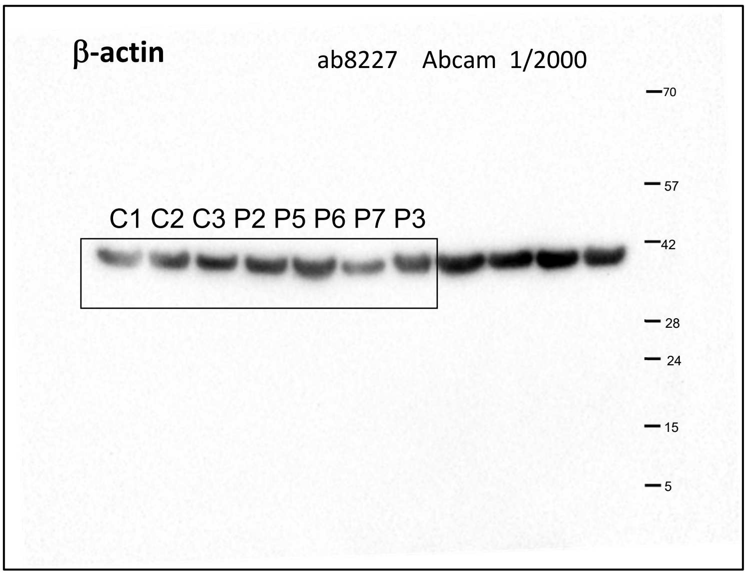
β-actin
ab8227 Abcam 1/2000
C1 C2 C3 P2 P5 P6 P7 P3
70
57
42
28
24
15
5

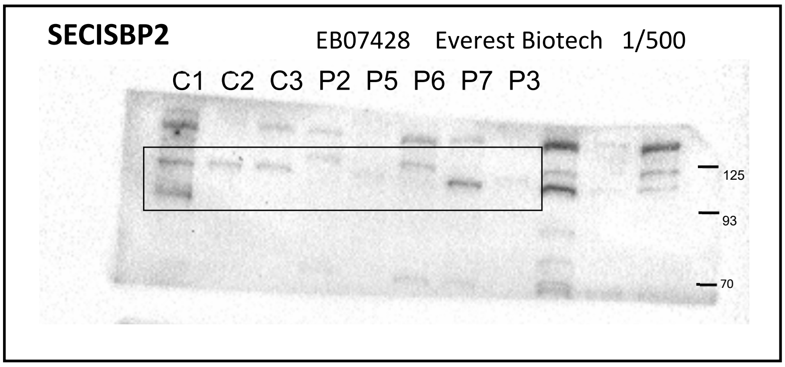
SECISBP2
EB07428 Everest Biotech 1/500
C1 C2 C3 P2 P5 P6 P7 P3
125
93
70

Supplementary Fig. 5B: Selenoprotein deficiency in patient P4.

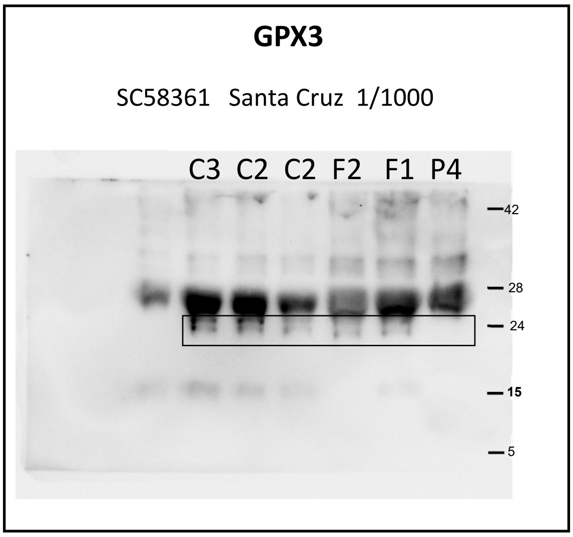

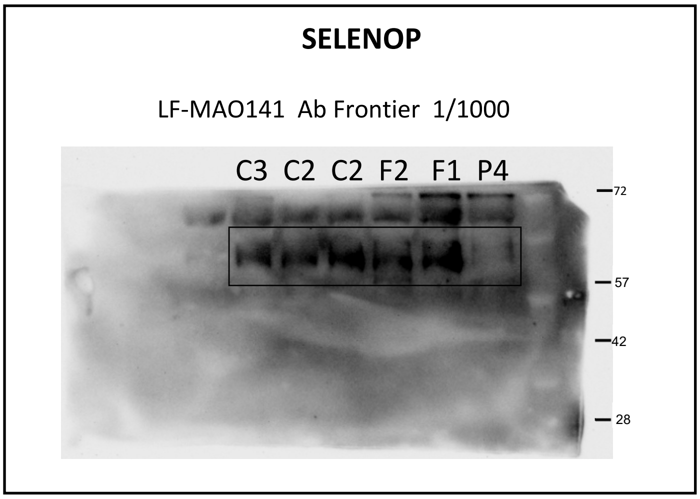

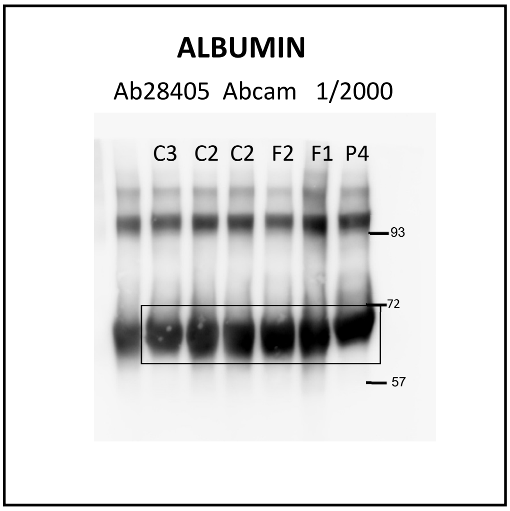

Supplementary Fig. 5C: Selenoprotein deficiency in patient P4.

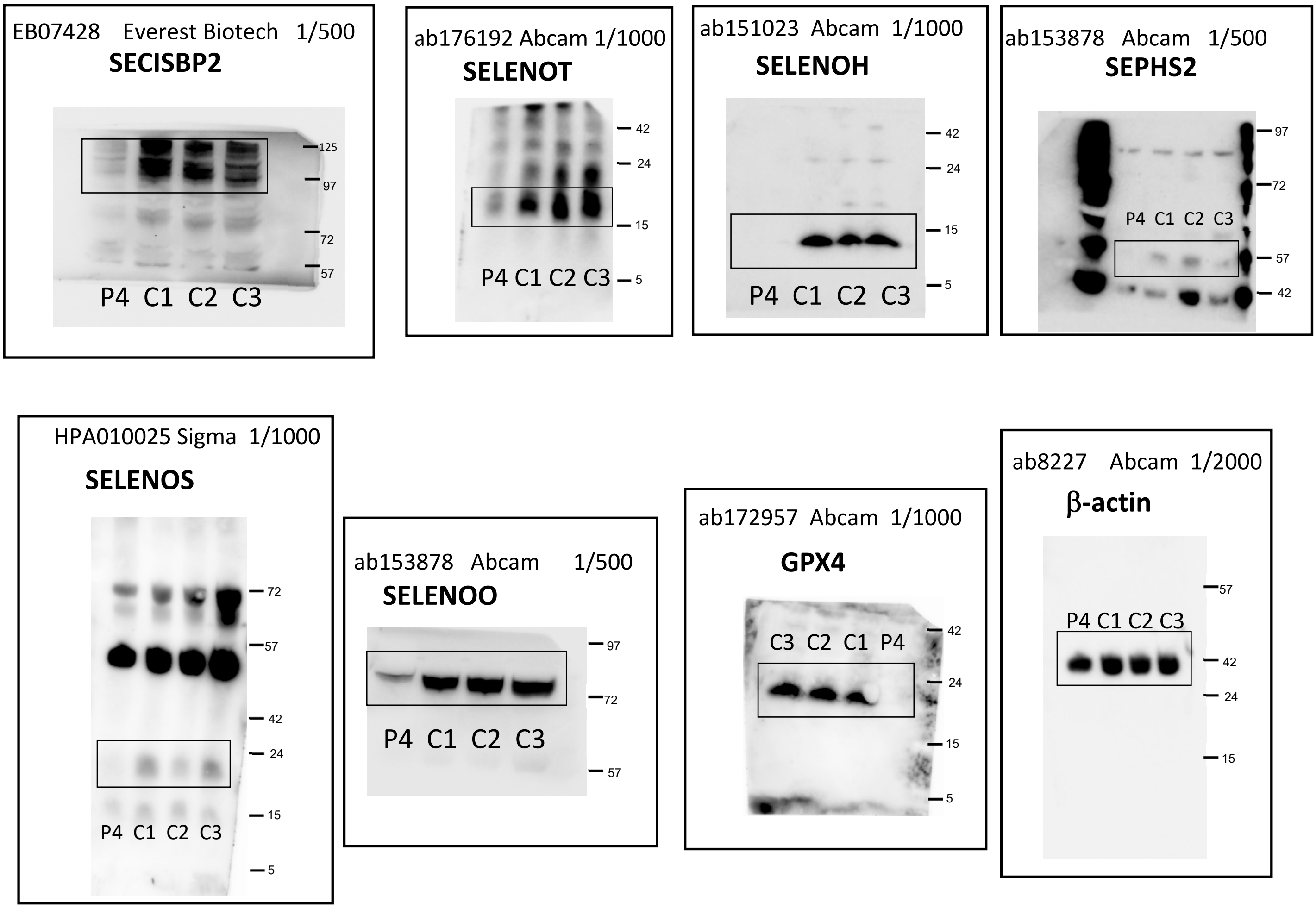

Supplementary Fig. 7D: Selenoprotein deficiency in *Secisbp*$^{Q333X/Q333X}$ mutant zebrafish.

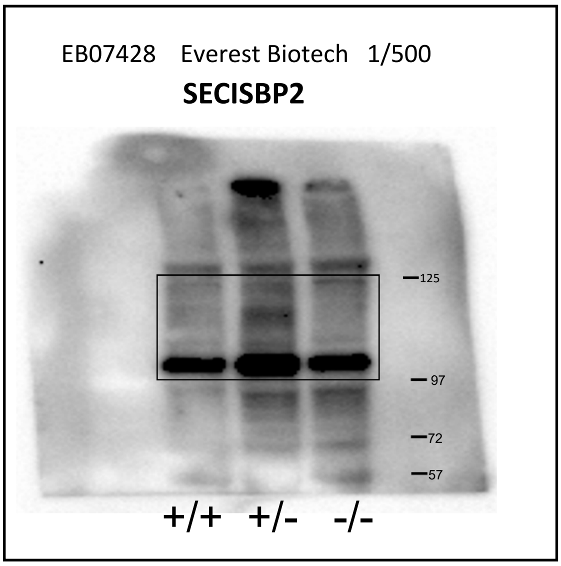

Supplementary Fig. 7F: Selenoprotein deficiency in *Secisbp*$^{Q333X/Q333X}$ mutant zebrafish.

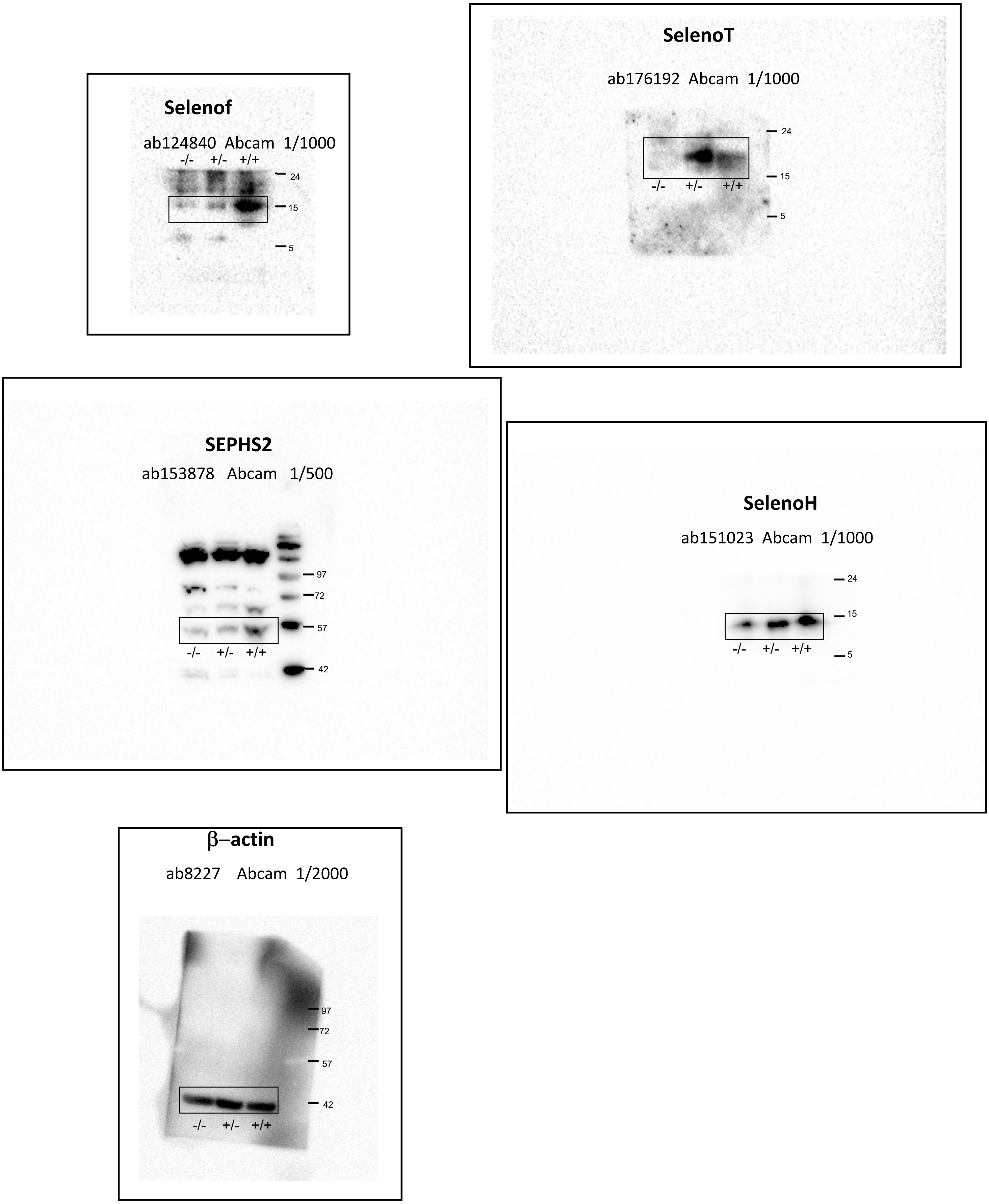

Supplementary Fig. 8C: Selenoprotein deficiency in Secisbp2 knockdown zebrafish.

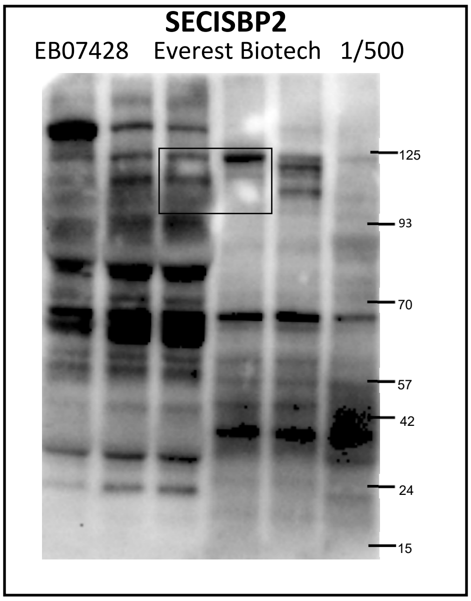

Supplementary Fig. 8E: Selenoprotein deficiency in Secisbp2 knockdown zebrafish.

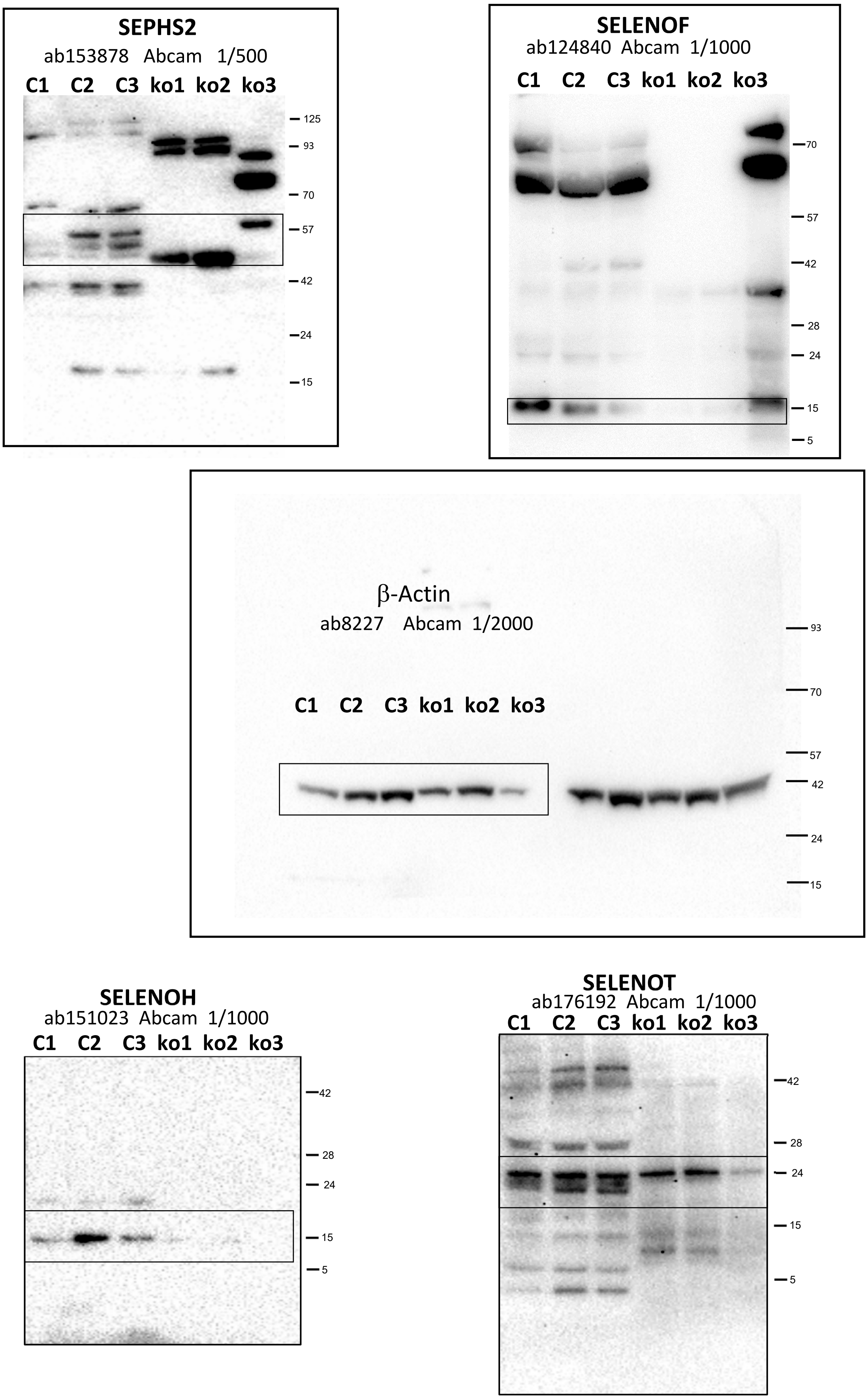

Supplement: Supplementary file 1 — Supplementary Information [file 41467_2023_43851_MOESM1_ESM.pdf]
