# Supplementary material for: Selenoprotein deficiency disorder predisposes to aortic aneurysm formation
Source: Nat Commun. 2023 Dec 2;14:7994. doi: 10.1038/s41467-023-43851-6 (PMC10693596; doi:10.1038/s41467-023-43851-6)
Supplement: Supplementary file 3 — Reporting Summary [file 41467_2023_43851_MOESM3_ESM.pdf]

nature portfolio

Corresponding author(s): Krishna Chatterjee

Last updated by author(s): Nov 21, 2023

Reporting Summary

Nature Portfolio wishes to improve the reproducibility of the work that we publish. This form provides structure for consistency and transparency in reporting. For further information on Nature Portfolio policies, see our Editorial Policies and the Editorial Policy Checklist.

Statistics

For all statistical analyses, confirm that the following items are present in the figure legend, table legend, main text, or Methods section.

n/a	Confirmed	
☐	☒	The exact sample size (*n*) for each experimental group/condition, given as a discrete number and unit of measurement
☐	☒	A statement on whether measurements were taken from distinct samples or whether the same sample was measured repeatedly
☐	☒	The statistical test(s) used AND whether they are one- or two-sided<br>*Only common tests should be described solely by name; describe more complex techniques in the Methods section.*
☐	☒	A description of all covariates tested
☐	☒	A description of any assumptions or corrections, such as tests of normality and adjustment for multiple comparisons
☐	☒	A full description of the statistical parameters including central tendency (e.g. means) or other basic estimates (e.g. regression coefficient) AND variation (e.g. standard deviation) or associated estimates of uncertainty (e.g. confidence intervals)
☐	☒	For null hypothesis testing, the test statistic (e.g. *F*, *t*, *r*) with confidence intervals, effect sizes, degrees of freedom and *P* value noted<br>*Give P values as exact values whenever suitable.*
☒	☐	For Bayesian analysis, information on the choice of priors and Markov chain Monte Carlo settings
☒	☐	For hierarchical and complex designs, identification of the appropriate level for tests and full reporting of outcomes
☒	☐	Estimates of effect sizes (e.g. Cohen's *d*, Pearson's *r*), indicating how they were calculated

Our web collection on statistics for biologists contains articles on many of the points above.

Software and code

Policy information about availability of computer code

Data collection	Clinical data was collected and entered into the case report form by members of the study team.<br>Flow cytometry data was collected with BD Accuri C6 Plus software.<br>Applied Biosystems 7900HT Fast Real-Time PCR System Software was used to collect qPCR data<br>Whole-exome sequencing : Illumina HiSeq2000 sequencer with TruSeq V3 chemistry<br>To identify carriers of SECISBP2 variants we utilized the exome sequencing data, generated as described elsewhere (https://www.ukbiobank.ac.uk/media/najcnoaz/access_064-uk-biobank-exome-release-faq_v11-1_final-002.pdf; https://biobank.ndph.ox.ac.uk/showcase/ukb/docs/UKB_WES_Protocol.pdf).<br>Zebrafish images:<br>Optical images were acquired using Leica Steromicroscope M205FA equipped with Leica DFC450FC digital camera and LAS V4.12 software.<br>Human vascular smooth muscle cell images were accquired using the Leica SP8 laser scanning confocal fluorescence microscope (Leica Microsystems) coupled to a white light laser (WLL), using acquisition software Leica Application Suite X (LAS X).<br>Confocal images were acquired with Nikon Eclipse Ti microscope (objective 20x) and NIS-element C2+ software.<br>Aorta images: sections were captured using a bright-field microscope with Image-Pro Insight 9.1 (Media Cybernetics, MD, USA).<br>Mitochondria lipid peroxidation in VSMC images were acquired using using the Opera Phoenix (Perkin Elmer).
Data analysis	Flow cytometry data was analysed with BD Accuri C6 Plus software.<br>GraphPad Prism Version 9.1.2 (GraphPad Software, San Diego, California USA, www.graphpad.com) was used to generate and analyse graphs.<br>ImageJ and GIMP-2.10 were used to analyse Western blotting. 4Peaks software DNA sequence viewer.<br>Applied Biosystems 7900HT Fast Real-Time PCR System Software was used to analyse qPCR data.<br>Whole-exome sequencing: UnifiedGenotyper, GATK v2.7.4 and ANNOVAR (version 2014-07-14)

Aortic traits in carriers of heterozygous, loss-of-function, SECISBP2 variants in UK Biobank: Variants were annotated using the Loss-of-Function Transcript Effect Estimator (LOFTEE8) plug-in implemented in the Variant Effect Predictor (VEP; v.105)9 (https://github.com/konradjk/loftee).
Zebrafish images: Images analysis and quantification were performed with ImageJ 1.52k. Adjustments of brighteness, contrast, resolution and TIFF compression were done using Adobe Photoshop CC 2015.5 .
Aorta images: sections were analysed using Image-Pro Insight 9.1 (Media Cybernetics, MD, USA).
Mitochondria lipid peroxidation in VSMC images were analysed using Harmony 4.9 Software (PerkinElmer).

For manuscripts utilizing custom algorithms or software that are central to the research but not yet described in published literature, software must be made available to editors and reviewers. We strongly encourage code deposition in a community repository (e.g. GitHub). See the Nature Portfolio guidelines for submitting code & software for further information.

Data

Policy information about availability of data

All manuscripts must include a data availability statement. This statement should provide the following information, where applicable:

- Accession codes, unique identifiers, or web links for publicly available datasets
- A description of any restrictions on data availability
- For clinical datasets or third party data, please ensure that the statement adheres to our policy

The genome sequencing data generated in this study are available under restricted access for patient privacy. Sequencing data can only be used to interrogate thyroid or aortopathy-related genes due to the nature of the consent obtained from participants. The processed sequencing data are available upon request from K. Chatterjee (kkc1@medschl.cam.ac.uk), with the period for response to the access request of one calendar month. Access to the UK Biobank genotype and phenotype data are open to all approved health researchers, accessible through https://www.ukbiobank.ac.uk. All other data supporting the findings described in this manuscript are available in the article, Supplementary Information or source data file. Source data are provided with this paper.

Research involving human participants, their data, or biological material

Policy information about studies with human participants or human data. See also policy information about sex, gender (identity/presentation), and sexual orientation and race, ethnicity and racism.

Reporting on sex and gender	The sex of patients was based on biological criteria, with gender being self-reported.
Reporting on race, ethnicity, or other socially relevant groupings	Three patients (P1, P2, P4) were (self-reported and researcher classified) as Caucasian and one patient (P4) was of African (self-reported and researcher classified) origin.
Population characteristics	All patients found to have mutations in SECISBP2, together with available, first-degree relatives were invited to participate. The participants were selected due to having a rare disorder caused by a mutation in SECISBP2 and all available family members with that mutation have been included. No other covariates like religion, age, socio-economic status etc have been considered for categorization.
Recruitment	Only patients with selenoprotein deficiency due to mutations in SECISBP2 and their available first degree relatives were invited to participate and all eligible individuals accepted the invitation. Travel costs of participants were reimbursed. No other financial compensation for participation was provided.
Ethics oversight	All studies in patients and control subjects, including analyses of participant-derived cells and tissues, were approved by Local Research Ethics Committees (Cambridgeshire Local Research Ethics Committee, LREC 98/154; Medical Ethics Committee, Erasmus MC, MEC-2015-362) and undertaken with prior written, informed consent of participants and/or parents. The authors affirm that human research participants or their parents/legal guardians provided written informed consent for publication of the potentially identifiable medical data included in this article.

Note that full information on the approval of the study protocol must also be provided in the manuscript.

Field-specific reporting

Please select the one below that is the best fit for your research. If you are not sure, read the appropriate sections before making your selection.

☒ Life sciences ☐ Behavioural & social sciences ☐ Ecological, evolutionary & environmental sciences

For a reference copy of the document with all sections, see nature.com/documents/nr-reporting-summary-flat.pdf

Life sciences study design

All studies must disclose on these points even when the disclosure is negative.

Sample size	For all animal studies, the minimal sample size was predetermined by the nature of experiments.<br>Sample size for human participants was not calculated a priori, with all available individuals with a mutation in SECISBP2 mutation being included.<br>For molecular experiments, no sample size calculation was performed, the results were collected from at least three biological replicates, or more as described in figure legends to ensure consistent phenotypes and to perform statistical analyses. For some experiments involving a

standard diagnostic procedure (eg. tissue histology) or with limited sample availability (eg primary human or mouse samples) only a single determination was undertaken or possible.

Data exclusions

No data was excluded

Replication

In assays from cells and tissues, results reported represent findings from multiple experiments (at least three independent occasions), with consistent replication of findings. Whenever possible, laboratory data generated from patients involved multiple determinations from samples taken on independent occasions.

Randomization

As an observational and mechanistic study, randomisation was not required

Blinding

Patients with selenoprotein deficiency were investigated (clinical, genetic, radiological, biochemical, pathological) prospectively, with comparison of quantitative changes in parameters against validated, normative, healthy population datasets. As some of the human data collected was under the auspices of clinical diagnosis or management of patients, blinding of investigators was not possible.

For animal studies the investigators were blinded to group allocation during data collection and analysis.

Reporting for specific materials, systems and methods

We require information from authors about some types of materials, experimental systems and methods used in many studies. Here, indicate whether each material, system or method listed is relevant to your study. If you are not sure if a list item applies to your research, read the appropriate section before selecting a response.

Materials & experimental systems

n/a	Involved in the study
☐	☒ Antibodies
☐	☒ Eukaryotic cell lines
☒	☐ Palaeontology and archaeology
☐	☒ Animals and other organisms
☐	☒ Clinical data
☒	☐ Dual use research of concern
☒	☐ Plants

Methods

n/a	Involved in the study
☒	☐ ChIP-seq
☐	☒ Flow cytometry
☒	☐ MRI-based neuroimaging

Antibodies

Antibodies used

Protein; Catalogue Number; Manufacturer
GPX1 ab108427 Abcam
GPX3 SC58361 Santa Cruz
GPX4 sc50497 Santa Cruz
SEPHS2 ab1538789 Abcam
Actin ab8227 Abcam
SECISBP2 EB07428 Everest Biotech
SELENOO ab172957 Abcam
SELENOS HPA010025 Sigma
SELENOH ab151023 Abcam
SELENOT ab176192 Abcam
SELENOI H00085465-A01 abnova
SELENOP LF-MAO141 Ab Frontier
SELENON Gift from P Guicheney (INSERM, UMR S956, Pitié-Salpêtrière Hospital, Paris, France)
SELENOF ab124840 Abcam
anti-8-oxo-G N45.1 Jaica
anti-g-H2AX 26350 Abcam
alpha-SMA M0851 Dako
anti-CD68 M0814 Dako
anti-Mac3 553322 BD Pharmingen
Albumin Ab28405 Abcam
Anti-rabbit IgG 7074 cell signaling
anti-Mouse IgG 31430 invitrogen
Anti-Goat/Sheep IgG A9452 Sigma-Aldrich
anti-calponin C2687, SIGMA
anti-alpha-SMA Ab5694, Abcam
anti-rabbit alexa-647 4414, Cell Signaling
anti-mouse alexa-488 A21202, invitrogen

Validation

All antibodies were validated both by commercial suppliers and in previous published work by the listed authors in accordance with in house protocols using appropriate positive and negative controls. Detailed information can be found on commercial websites.

GPX1 ab108427 Abcam:

https://www.abcam.com/products/primary-antibodies/glutathione-peroxidase-1-antibody-epr3312-ab108427.html
GPX3 SC58361 Santa Cruz
https://www.scbt.com/p/gpx-3-antibody-23b1
GPX4 sc50497 Santa Cruz
https://www.scbt.com/p/gpx-4-antibody-h-90?requestFrom=search
SEPHS2 ab153878 Abcam
https://www.abcam.com/products/primary-antibodies/selenophosphate-synthetase-2-antibody-ab153878.html
Beta Actin ab8227 Abcam
https://www.abcam.com/products/primary-antibodies/beta-actin-antibody-ab8227.html
SECISBP2 EB07428 Everest Biotech
https://everestbiotech.com/product/goat-anti-sbp2-antibody/
SELENOO ab172957 Abcam
https://www.abcam.com/products/primary-antibodies/selo-antibody-epr11968-ab172957.html
SELENOS HPA010025 Sigma
https://www.sigmaaldrich.com/GB/en/product/sigma/hpa010025
SELENOH ab151023 Abcam
https://www.abcam.com/products/primary-antibodies/selh-antibody-ab151023.html
SELENOT ab176192 Abcam
https://www.abcam.com/products/primary-antibodies/selt-antibody-n-terminal-ab176192.html
SELENOI H00085465-A01 abnova
https://www.novusbio.com/products/seli-antibody_h00085465-a01
SELENOP LF-MAO141 Ab Frontier
https://uk.vwr.com/store/product/15359885/anti-sepp1-mouse-monoclonal-antibody-clone-37a1
SELENON
Human Molecular Genetics, Volume 12, Issue 9, 1 May 2003, Pages 1045–1053, https://doi.org/10.1093/hmg/ddg115
SELENOF ab124840 Abcam
https://www.abcam.com/products/primary-antibodies/sep15-antibody-ncir128a-ab124840.html
anti-8-oxo-G N45.1 Jaica
https://www.jaica.com/e/products_dna_8ohdg_ab.html
anti-gamma-H2AX 26350 Abcam
https://www.abcam.com/products/primary-antibodies/gamma-h2ax-phospho-s139-antibody-9f3-ab26350.html
alpha-SMA M0851 Dako
https://www.agilent.com/en/product/immunohistochemistry/antibodies-controls/primary-antibodies/actin-%28smooth-muscle%29-%28concentrate%29-76542
anti-CD68 M0814 Dako
https://www.agilent.com/en/product/immunohistochemistry/antibodies-controls/primary-antibodies/cd68-%28concentrate%29-76535
anti-Mac3 553322 BD Pharmingen
https://www.bdbiosciences.com/en-us/products/reagents/western-blotting-and-molecular-reagents/purified-rat-anti-mouse-cd107b.553322
Albumin Ab28405 Abcam
https://www.abcam.com/products/primary-antibodies/human-serum-albumin-antibody-1g2-ab28405.html
Albumin Ab28405 Abcam
https://www.abcam.com/products/primary-antibodies/human-serum-albumin-antibody-1g2-ab28405.html

Anti-rabbit IgG 7074 cell signaling
https://www.cellsignal.com/products/secondary-antibodies/anti-rabbit-igg-hrp-linked-antibody/7074
anti-Mouse IgG 31430 invitrogen
https://www.thermofisher.com/order/genome-database/dataSheetPdf?productype=antibody&productsubtype=antibody_secondary&productId=31430&version=343
Anti-Goat/Sheep IgG A9452 Sigma-Aldrich
https://www.sigmaaldrich.com/GB/en/product/sigma/a9452

anti-calponin C2687, SIGMA
https://www.sigmaaldrich.com/deepweb/assets/sigmaaldrich/product/documents/208/639/c2687dat.pdf
anti-alpha-SMA Ab5694, Abcam
https://www.abcam.com/products/primary-antibodies/alpha-smooth-muscle-actin-antibody-ab5694.html
anti-rabbit alexa-647 4414, Cell Signaling
https://www.cellsignal.com/products/secondary-antibodies/anti-rabbit-igg-h-l-f-ab-2-fragment-alexa-fluor-647-conjugate/4414
anti-mouse alexa-488 A21202, invitrogen
https://www.thermofisher.com/antibody/product/Donkey-anti-Mouse-IgG-H-L-Highly-Cross-Adsorbed-Secondary-Antibody-Polyclonal/A-21202

Eukaryotic cell lines

Policy information about cell lines and Sex and Gender in Research

Cell line source(s)	The primary dermal fibroblast and vascular smooth muscle cell lines were generated from skin biopsies or tissue resected at aortic surgery respectively from male patients and male healthy controls, with their prior written informed consent according to ethically-approved protocols.
Authentication	All cell lines were shown to contain SECISBP2 mutations by DNA sequencing, with functional studies confirming deficiency of selenocysteine-containing proteins. Quantitative PCR and immunohistochemistry verified expression of vascular smooth muscle specific markers in VSMCs.

Mycoplasma contamination | The dermal fibroblast lines tested negative for mycoplasma.

Commonly misidentified lines (See ICLAC register) | No commonly misidentified cell lines were used.

Animals and other research organisms

Policy information about studies involving animals; ARRIVE guidelines recommended for reporting animal research, and Sex and Gender in Research

Laboratory animals | Mice were housed at 22C, with 40-60% humidity and a 12 hour light/dark cycle. All mice used (Wild type and Myh11-CreERt2) have a C57BL/6N background and tissues were harvested from 13 week old male mice.
The zebrafish Secisbp2 Q333X mutant line (allele designation sa33758, Tuebingen/AB strain), generated by the Zebrafish Mutation Project at the Sanger Institute is available from European (EZRC) or International (ZIRC) resource centres. Zebrafish used for morpholino experiments have a Tuebingen/AB strain background.

Wild animals | Study did not involve wild animals.

Reporting on sex | Myh11-CreERt2 is Y-linked, so all experiments were performed using males as described in the Methods section.
Zebrafish data has not been collected.

Field-collected samples | Study did not involve samples collected in field.

Ethics oversight | As stated in the Methods, zebrafish (Danio rerio) embryos obtained from natural spawning were raised and maintained according to EU regulations on laboratory animals (Directive 2010/63/EU). The zebrafish studies were approved by the Body for the protection of Animals (OPBA) of the University of Milan, Italy (protocol 198283). Depending on the experiments, fish were anesthetized or euthanized with buffered tricaine solution (MS-222, Sigma Aldrich) at 16mg/L or 300mg/L, respectively.

As stated in the Methods, VSMC-targeted Secisbp2-deficient and control mice were studied following local ethical approval (UK Home Office Project license P452C9545). Animals were sacrificed using carbon dioxide after 28 days of Angiotensin II infusion, unless they reached a non-humane endpoint or died before.

Note that full information on the approval of the study protocol must also be provided in the manuscript.

Clinical data

Policy information about clinical studies
All manuscripts should comply with the ICMJE guidelines for publication of clinical research and a completed CONSORT checklist must be included with all submissions.

Clinical trial registration | This study comprises identification of aortic dilatation by clinical surveillance of patients with selenoprotein deficiency and investigation of the pathogenesis of this phenotype in selenoprotein-deficient cells and tissues from patients or animal models.

Study protocol | Not applicable

Data collection | Not applicable

Outcomes | Not applicable

Flow Cytometry

Plots

Confirm that:

☒ The axis labels state the marker and fluorochrome used (e.g. CD4-FITC).

☒ The axis scales are clearly visible. Include numbers along axes only for bottom left plot of group (a 'group' is an analysis of identical markers).

☒ All plots are contour plots with outliers or pseudocolor plots.

☒ A numerical value for number of cells or percentage (with statistics) is provided.

Methodology

Sample preparation | VSMC or dermal fibroblast membrane lipid peroxidation was measured after loading 30min with 1 µM BODIPY 581/591 C11 (ThermoFisher Scientific) with fluorescence monitored by flow cytometry on channels FL1-H at 530 nm and FL2-H at 585 nm.
Annexin V positivity of VSMCs was measured using a FITC-Annexin V apoptosis detection kit (BD Biosciences) following the manufacturer’s protocol.
H_2O_2 (0, 50, 100 or 250 µM) induced membrane lipid peroxidation in Ficoll purified PBMCs was measured after loading the

	cells for 30 min with 1 µM BODIPY 581/591 C11 (ThermoFisher Scientific) with fluorescence monitored by flow cytometry on channels FL1-H at 530 nm and FL2-H at 585 nm.
Instrument	BD Accuri C6 Plus
Software	Flow cytometry data was collected and analysed with BD Accuri C6 Plus software.
Cell population abundance	No sorting was peformed
Gating strategy	FSC/SSC gating on main viable cells population and gates for markers were set in relation to controls.

☒ Tick this box to confirm that a figure exemplifying the gating strategy is provided in the Supplementary Information.
